# Enhancing Drug-Target Interaction Prediction through Transfer Learning from Activity Cliff Prediction Tasks

**DOI:** 10.1021/acs.jcim.5c00484

**Published:** 2025-06-30

**Authors:** Regina Ibragimova, Dimitrios Iliadis, Willem Waegeman

**Affiliations:** Department of Data Analysis and Mathematical Modelling, 26656 Ghent University, Coupure Links, Ghent 9000, Belgium

## Abstract

Recently, machine learning (ML) has gained popularity in the early stages of drug discovery. This trend is unsurprising given the increasing volume of relevant experimental data and the continuous improvement of ML algorithms. However, conventional models, which rely on the principle of molecular similarity, often fail to capture the complexities of chemical interactions, particularly those involving activity cliffs (ACs)—compounds that are structurally similar but exhibit evidently different activity behaviors. In this study, we explore whether transfer learning from AC prediction can enhance prediction of interactions between drug-like compounds and protein targets. We develop a universal model for AC prediction and investigate its impact when transferring learned representations to DTI prediction. Our results suggest that AC-informed transfer learning has the potential to improve the handling of challenging AC-related scenarios, while maintaining overall predictive performance. This study contributes to the ongoing exploration of strategies to enhance ML-based DTI prediction, particularly in cases where conventional approaches face limitations.

## Graphical abstract

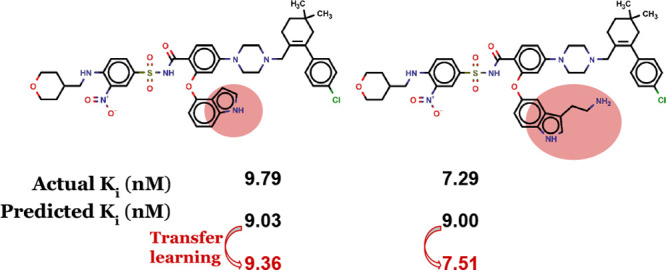

## 1 Introduction

Drug development is a challenging, lengthy, and costly process. Although the application of artificial intelligence is creating new opportunities throughout the entire cycle of the drug development process, there is evidence that one of the primary reasons for the high failure rates of drug candidates during clinical development has been a lack of clinical efficacy.^1^ Drug-target interaction (DTI) prediction, aided by computational methods, offers a more rapid and cost-effective alternative.^2^ In this work, the term drug refers to small, bioactive compounds with potential therapeutic effects, which may not necessarily represent approved or final drug candidates. Moreover, the application of machine learning (ML) in drug–drug interaction and DTI has increased due to the abundance of accessible data, the power of the available tools and services, and the growing demand brought on by high-throughput approaches.^3^ Recent studies have further demonstrated the effectiveness of pretrained representations and network-based learning for improving DTI predictions across diverse biomedical domains.^4,5^ These developments reflect a broader shift toward more sophisticated, data-driven strategies in computational drug discovery.

Studies on DTI prediction have demonstrated that models with good performance can be constructed by utilizing various optimization or calculation techniques during the phases of data set acquisition, feature extraction and processing, and task algorithm selection.^6^ Most of the methods are based on the principle of similarity, which states that similar compounds have similar properties and share similar targets.^7^ One of the most widely used frameworks defines the prediction of DTIs as a classification problem and formulates various types of similarity functions as input.^6,8^ However, there is a known weakness referred to as activity cliffs (ACs).^9^ Even when a model has a high overall predictive performance, it may struggle in the case of ACs.^10–12^

Predicting ACs is challenging for ML due to three primary problems. First, in ACs, small structural changes in compounds can lead to dramatic changes in activity, resulting in highly discontinuous structure–activity relationships.^10^ This phenomenon has two sides. On the one hand, it accelerates drug research by providing important information for drug design and studying drug–drug and drug–target interactions.^9^ On the other hand, it may lead to the unexpected loss of desired properties.^13^ Capturing such cases can be difficult for ML models, potentially leading to a decrease in performance.^10,13^ Second, data sets for ACs and non-ACs are highly imbalanced.^14^ Lastly, predictions should be made at the level of compound pairs, in contrast to the usual molecular property predictions, which are made for individual compounds.^14^

Despite achieving reasonable overall performance, models often struggle, especially in predicting ACs. While there are examples in the literature of attempts to predict ACs,^12,14–17^ the subsequent application of these predictions to enhance DTI model performance has not yet been extensively explored.^10^ Although ACs are frequently seen as being problematic due to the challenges they pose, they also encode essential information for applications like structure–activity connection analysis^18^ and hit-to-lead optimization.^19^ In fact, models capable of capturing the complexity of ACs may offer a more accurate representation of the structure–activity landscape.^11^ Therefore, the explicit integration of AC-informed modeling into DTI frameworks—particularly through transfer learning—remains an underexplored area that could lead to more robust and generalizable models.

Integrating such awareness via transfer learning could potentially boost performance in this domain, thereby improving overall model performance. Deep learning models, however, require large data sets for effective training. Traditional ML approaches rely on training and testing data sets with identical input feature spaces. Nevertheless, large volumes of data are often unavailable for certain compounds or targets due to technical difficulties or high costs, resulting in a lack of models for these cases.^20^ Transfer learning presents a promising approach to this challenge. It mitigates this issue by pretraining a model on one task and transferring the knowledge to a related, yet distinct, task.^21^ However, it remains an open question how well AC-informed transfer learning can improve DTI prediction.

The objective of this research is to investigate whether knowledge gained from AC prediction can enhance the robustness and accuracy of DTI models. Specifically, we explore whether transfer learning can improve model generalization in challenging cases where ACs are present. We do not claim that this is the best or only approach to improving DTI prediction but rather seek to assess its potential as a complementary strategy. Additionally, we examine how transfer learning may mitigate challenges associated with data scarcity in DTI prediction by leveraging pretrained representations from the AC task. By addressing these questions, we aim to contribute to a better understanding of the relationship between AC-aware modeling and DTI prediction in the context of computational drug discovery.

This paper is organized as follows: we first describe the methodology for the AC prediction and DTI prediction tasks. Subsequently, we discuss the results of our experimental study, organized into subsections where we analyze different aspects of the experiments. We examine the impact of transferring encoders and evaluate model performance in AC-related scenarios of increasing difficulty. The findings highlight the potential of AC-informed transfer learning to enhance DTI prediction in drug discovery workflows.

## 2 Methodology

### 2.1 Data Sets and Data Preprocessing

The KIBA^22^ and BindingDB^23^ data sets were utilized in this study because of their varying numbers of drugs, targets, and measured affinities as well as their popularity in DTI prediction publications. Their brief statistics are shown in Table 1.

**Table 1:** Overview of the Datasets

data set	# drugs	# targets	# DTIs	# ACs (%)	# non-ACs (%)
KIBA	2068	229	118,254	9916 (12%)	70,601 (88%)
BindingDB (*K*_*i*_)	32,347	1018	78,628	71,304 (28%)	181,499 (72%)

We define two compounds as an AC pair if: (1) the compounds are structurally similar; (2) the compounds have different affinities toward the same target. There is no one strict definition of how a similarity should be computed, and several possible methods can be used. The most popular one is the matched molecular pair,^14^ which pairs molecules that only differentiate by a single chemical modification at a specific site.^24^ Another approach is to consider structural similarities and differences in a pairwise manner, using several criteria, such as substructure, scaffold, or SMILES similarity.^12^ Based on previous research,^12^ we defined a pair of compounds as an AC if the compounds were at least 90% similar according to any of the three criteria mentioned above and the affinities of the compounds toward the same target showed at least a 10-fold difference.

To identify AC pairs, the data set was preprocessed through several steps. First, for each target, drugs that interact with the target were paired based on their structural similarity. Pairs with a similarity of over 90%, according to at least one criterion mentioned above, were then selected. Next, for each selected pair, the differences in drug affinities toward the target were calculated. If the difference in affinities exceeded a predefined affinity threshold, the pair was classified as an AC pair. Otherwise, the pair was classified as a non-AC pair.

The study focuses on two tasks: AC and DTI prediction. While improving DTI prediction is the primary objective, AC problem is leveraged as an auxiliary task to enhance the model’s performance. In the preprocessing step for both AC and DTI tasks, Extended Connectivity Fingerprints (ECFPs)^25^ were generated from the molecules encoded in SMILES^26^ format using RDKit and used as input features. Proteins were assigned unique labels and transformed to a simple one-hot encoded vector. The length of the vector was equal to the total number of unique proteins in the data set and all the positions were set to zero, except from the one that mapped to the unique protein and was set to one. While one-hot encoding does not incorporate structural or sequential information, it was intentionally selected in this work to minimize complexity and focus on the ligand-side representation learning. Encoding proteins using modern deep learning methods typically requires extensive pretraining on large protein corpora, which falls outside the scope of this study. Given the limited protein diversity in the data set, one-hot encoding also helps reduce the risk of overfitting. This design choice allows us to more directly evaluate the impact of transfer learning strategies derived from the small molecule (ligand) domain.

### 2.2 Data Set Splitting Methods

There are several approaches to splitting the data in ML tasks, each affecting model performance and generalization differently.^27^ The data set can be split randomly, based on drugs, where each drug appears only in the training or test set, or based on targets, ensuring that each target (protein) is included exclusively in either the training or test set, but not both.^28^ Splitting can also involve a combination of these approaches.^28^ For the AC task, for example, one could have scenarios with either one novel compound or two novel compounds. While we did not explore all of these possibilities, they are certainly viable options for data splitting. We opted for the setting that we believe best reflects real-world challenges, but other configurations could be equally relevant depending on the certain task.

For both the AC and DTI tasks, two data set splitting methods were utilized: random splitting and compound-based splitting. In the random split, compound pairs were randomly assigned to the training and test sets. However, this approach may lead to overoptimistic results, as the same individual compounds could appear in both sets via different pairs, potentially leading to data leakage. In contrast, the compound-based split, as demonstrated in Figure 1, ensures that each compound is assigned to either the training or testing set exclusively, and no overlap of compounds occurs across the splits. By ensuring that the same compounds do not appear in different splits, the compound-based approach provides a more reliable and realistic assessment of the model’s performance. This approach prevents the model from memorizing specific compound properties, which might appear across different splits, and thus provides a more robust evaluation of its generalization. To ensure a fair and consistent evaluation, we retained the same training and test data sets for the DTI task. This approach guarantees that comparisons between the AC and DTI tasks are based on equivalent data partitions, and that any observed differences in performance are not due to differences in data splitting strategy.

**Figure 1:**
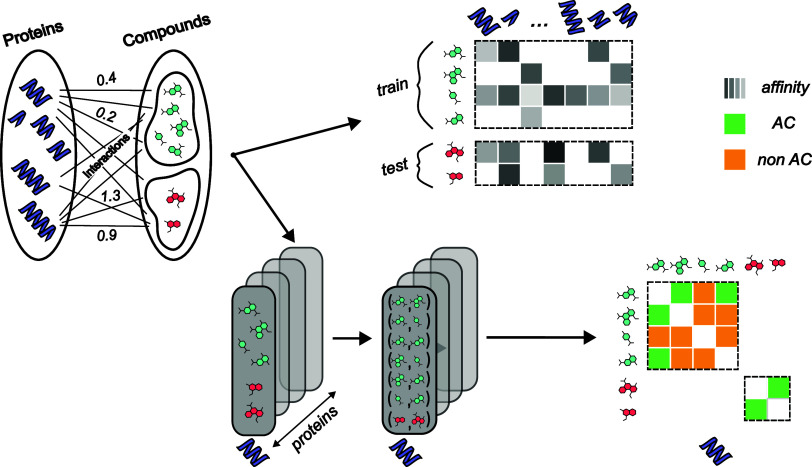
Schematic representation of the compound-based data set splitting process for the two main tasks considered in this study: DTI and AC prediction. Initially, the data set is split into training (green) and testing (red) sets. For the DTI task, drug-target pairs are directly generated within these sets. In contrast, the AC task involves three additional steps (shown at the bottom): first, identifying all drugs that interact with a specific protein in both the training and testing sets; second, pairing these drugs for each protein based on their structural similarity; and third, categorizing each pair as either an AC or non-AC based on predefined criteria. The labels in the figure indicate AC pairs in green, non-AC pairs in orange, and continuous affinity levels in grayscale.

As shown in Figure 1, the data set splitting approaches initially follow the same procedure for both the DTI and AC tasks, with drugs being divided into training (shown in green color) and testing sets (shown in red color) in a single step. This initial split of drugs ensures that the same compounds are consistently used across both tasks, thereby maintaining separate training and testing sets and avoiding any data leakage in subsequent steps.

For the DTI task, after the initial drug split, drug-target pairs are formed directly within the training and testing sets. However, for the AC task, additional steps are undertaken within the predefined training and testing sets to ensure accurate classification. Specifically, in step (a), within each training and testing set, all drugs that interact with a specific protein are selected. Then, in step (b), these selected drugs are paired based on their structural similarity, as outlined in Section 2.1. Pairs are evaluated for similarity and affinity differences to identify potential AC pairs. Finally, in step (c), the data sets for all targets are then combined within each training and testing set. Although this approach may result in fewer available pairs for the AC task, it is crucial for avoiding data leakage and ensuring a robust and reliable evaluation of the models. By consistently maintaining the same training and testing sets for both tasks, this method provides a solid foundation for accurately assessing the models’ performance without the risk of overfitting. It is worth noting that by combining data for all targets and using a unified model across these targets, we create a larger data set. This is beneficial for deep learning models, which typically require vast amounts of data to perform effectively.

### 2.3 Model Details

The architecture of the models consists of two branches, as commonly done in DTI prediction models.^29–33^ As shown in Figure 2, this design allows for the separate handling drug-related and target-related information, with specific encoders for each of them. It is worth noting that the protein encoder consists of a simple dense layer that maps the sparse one-hot encoded vector, to a dense representation compatible with the rest of the architecture. The embeddings produced by these encoders are aggregated to create a combined representation, which is then utilized to make a final prediction.

**Figure 2:**
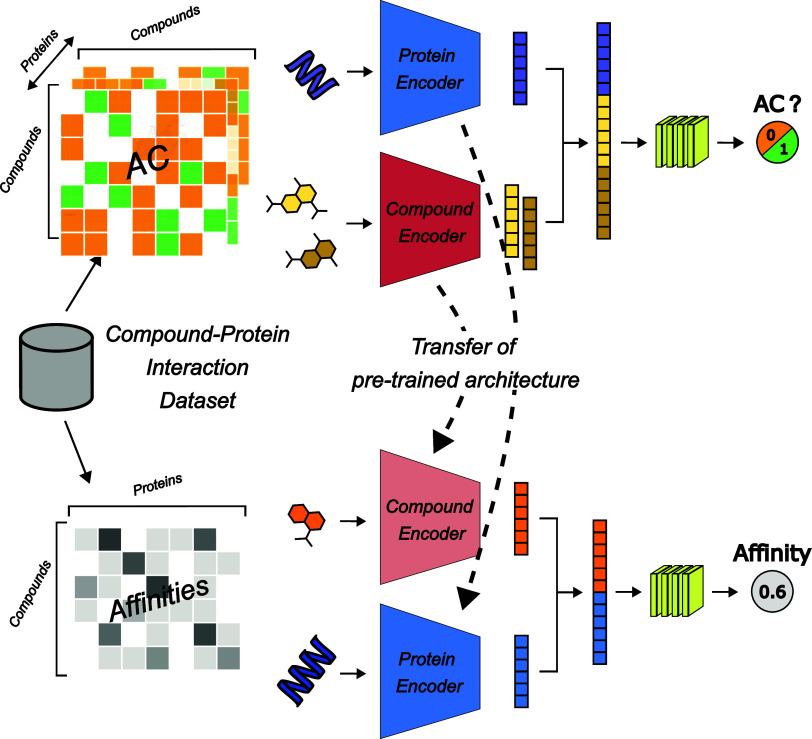
Schematic representation of the model architectures used for AC (top) and DTI (bottom) tasks. Both tasks employ separate encoders for drugs (Compound Encoder) and targets (Protein Encoder). In the AC task, the model processes both drugs through the same Compound Encoder, concatenates their embeddings with the target embedding from the Protein Encoder, and predicts whether the pair represents an AC (labeled as 1) or non-AC (labeled as 0) for a given target. For the DTI task, drug and target embeddings are concatenated to predict drug-target affinity as a continuous value, leveraging transferred features from the AC task to improve prediction accuracy for novel compound–protein interactions.

The ACs task is a binary classification problem, and the aim of the AC model is to predict whether a pair of the drugs given is AC (represented as 1) or non-AC (represented as 0) toward the given target. In the AC model, both drugs are processed through the same compound encoder to generate their respective embeddings, which are then used to assess the AC status. In contrast, the DTI model’s architecture, while also employing a two-branch structure, focuses on the interaction between a single drug and a target. Separate encoders are utilized for drugs (Compound Encoder) and targets (Protein Encoder). In this model, the aim is to predict the affinity or activity level of the drug toward the target, which is represented as a continuous value (e.g., 0.6). The model can leverage a pretrained architecture, transferring learned features from the AC task.

### 2.4 Experimental Setup and Hyper-Parameter Optimization

In this study, we tested various combinations of model configurations, data sets, and transfer learning strategies to evaluate the performance of the models under different conditions. Supplementary Table S1 provides an overview of the experimental setup, summarizing all combinations of data sets, splits, tasks, and training configurations used.

A random search was conducted to find the best configuration of the model hyper-parameters in both AC and DTI baseline tasks. For each problem, 100 configurations were evaluated, each with a maximum allowance of 100 epochs and early stopping on the validation loss. Details of the search space are provided in the Supporting Information 2. The Weights and Biases platform was used for logging,^34^ and the links are provided in Supplementary Table S1. The best configuration was trained three times to ensure consistency of the results, and evaluated using the molecules in the test set.

In the transfer learning models, a similar procedure was applied for both the warm starting and freezing weights settings. However, in the case where an additional layer was added to the weight-freezing model, the best parameters obtained from the weight-freezing setup were retained. Additional parameters related to the new layer were then varied using a grid search, resulting in a total of 42 configurations being evaluated.

### 2.5 Performance Measures

#### 2.5.1 AC Task

Given the imbalanced nature of this task, appropriate evaluation metrics are essential for assessing the model’s performance. In this work, the F1-score, Matthews Correlation Coefficient (MCC) and Balanced Accuracy were employed as the metrics to evaluate the model. The F1-score provides a balanced assessment of the model’s ability to predict ACs by calculating the harmonic mean of precision and recall. Precision represents how many of the predicted ACs are correct (eq 1), while recall (or true positive rate) measures the proportion of actual ACs that are correctly identified (eq 2). The F1-score combines both metrics, ensuring that the evaluation accounts for both false positives and false negatives, which is crucial in imbalanced data sets like the AC task (eq 3).

$$\mathrm{Precision}=\frac{\mathrm{TP}}{\mathrm{TP}+\mathrm{FP}}$$ 1

$$\mathrm{Recall}=\frac{\mathrm{TP}}{\mathrm{TP}+\mathrm{FN}}$$ 2

$$F1=2\times \frac{\mathrm{Recall}\times \mathrm{Precision}}{\mathrm{Recall}+\mathrm{Precision}}$$ 3

where TP, FP, and FN represent the total counts of true positive, false positive, and false negative predictions, respectively.

The MCC was included to ensure an effective evaluation of model performance in imbalanced data sets.^35^ MCC considers all prediction categories, including true negatives (TN), and provides a comprehensive measure of model performance across all classes:

$$\mathrm{MCC}=\frac{\mathrm{TP}\times \mathrm{TN}-\mathrm{FP}\times \mathrm{FN}}{\sqrt{\left(\mathrm{TP}+\mathrm{FP})\times (\mathrm{TP}+\mathrm{FN})\times (\mathrm{TN}+\mathrm{FP})\times (\mathrm{TN}+\mathrm{FN}\right)}}$$ 4

To further ensure robustness against class imbalance, Balanced Accuracy was included. It accounts for the ability to correctly classify both classes (AC and non-AC) by averaging the true positive rate (sensitivity) and the true negative rate (specificity):

$$\mathrm{Balanced}\;\mathrm{Accuracy}=\frac{1}{2}\left(\frac{\mathrm{TP}}{\mathrm{TP}+\mathrm{FN}}+\frac{\mathrm{TN}}{\mathrm{TN}+\mathrm{FP}}\right)$$ 5

#### 2.5.2 DTI Task

In order to evaluate the performance of the DTI task, macro- and microaveraging of well-known metrics was used:

- Microaveraging: this approach provides an evaluation of the model’s performance across the entire data set by treating all predictions as a single pool, regardless of the target. The microaveraged Root Mean Square Error (RMSE) is calculated by summing the squared differences between the observed and predicted values for all samples across all targets, dividing by the total number of samples, and then taking the square root:
RMSEmicro=∑t=1T∑i=1nt(yit−y^it)2∑t=1Tnt 6
  where *y*_*it*_ and *ŷ*_*it*_ are the true and predicted values for sample *i* of target *t*, respectively, *n*_*t*_ represents the number of samples for target *t*, and *T* is the total number of targets.
- Macroaveraging: this approach ensures that every target contributes equally to the performance metric, giving a balanced picture of the model’s performance across all targets. Here, RMSE is first calculated for each target individually, and then the average of these RMSE values across all targets is computed:
RMSEmacro=1T∑t=1T1nt∑i=1nt(yit−y^it)2 7

## 3 Results and Discussion

### 3.1 AC Task

It is important to note that ACs represent the minority class, making this an imbalanced problem, as outlined in Table 1. Although the AC task is an important aspect of the overall study, our primary focus remains on improving the DTI task, so we present the AC prediction results concisely. For both data sets, the parameters of the best AC models for random and compound-based splits are provided in Supplementary Table S2. The performance was assessed using a test set of molecules, yielding satisfactory results, as presented in Supplementary Table S3. In the case of the BindingDB random split, the Uniform Manifold Approximation and Projection visualizations^36^ of the input features and hidden states after the hidden layers are provided in Supplementary Figure S1.

The two-branch model structure in the AC tasks allowed us to aggregate data across all targets, effectively expanding the data set size and complexity. This approach is advantageous in deep learning, where substantial data typically enhances model performance. Leveraging data from multiple targets in a unified model enabled better learning of complex patterns and improved generalization, especially in cases where data is limited for individual targets. This structure ultimately enhanced both the robustness and accuracy of our models across diverse patterns.

### 3.2 DTI Prediction: Baseline Models

We evaluated AC-specific DTI prediction performance across varying levels of AC severity using heatmaps that adjust thresholds for compound similarity and affinity differences toward the same target. This approach provides a systematic and detailed visualization of the AC phenomenon compared to traditional ad hoc thresholding methods. It is important to emphasize that the AC phenomenon may not be evident when both AC and non-AC compounds are analyzed together due to the significantly higher number of non-ACs. By progressively filtering out structurally dissimilar compounds and concentrating on those with higher structural similarity, the AC effect becomes more pronounced, enabling for a clearer distinction between AC and non-AC compounds. This method is particularly advantageous in limited data scenarios, although extreme AC cases may lack representativeness due to the small number of compound pairs.

Supplementary Figures S2 and S3 present heatmaps showing the summed number of pairs for all targets at different thresholds in the test set of both data sets. In these heatmaps, each bin represents a combination of structural similarity and affinity difference thresholds. Specifically, a pair is assigned to a cell only if it meets both the similarity and affinity criteria. In contrast, pairs that do not meet both thresholds would be excluded from that bin and placed in others based on their characteristics (e.g., lower similarity or smaller affinity differences). Subgroups with fewer than 100 pairs were masked in gray to indicate insufficient data.

The hyperparameters of the best DTI models for both data sets under random and compound-based splits are shown in Supplementary Tables S4 and S5, respectively. The model’s performance on the test set for the filtered groups of the KIBA and BindingDB data sets for compound-based split is illustrated in Figure 3. In both data sets, the model demonstrates decent performance in groups containing both ACs and non-ACs (bottom left of the heatmaps). However, performance declines as non-ACs are progressively filtered out, with the model struggling to accurately predict interactions in groups with fewer non-ACs and more challenging AC cases (upper right areas of the heatmaps, where both affinity and similarity thresholds are high).

**Figure 3:**
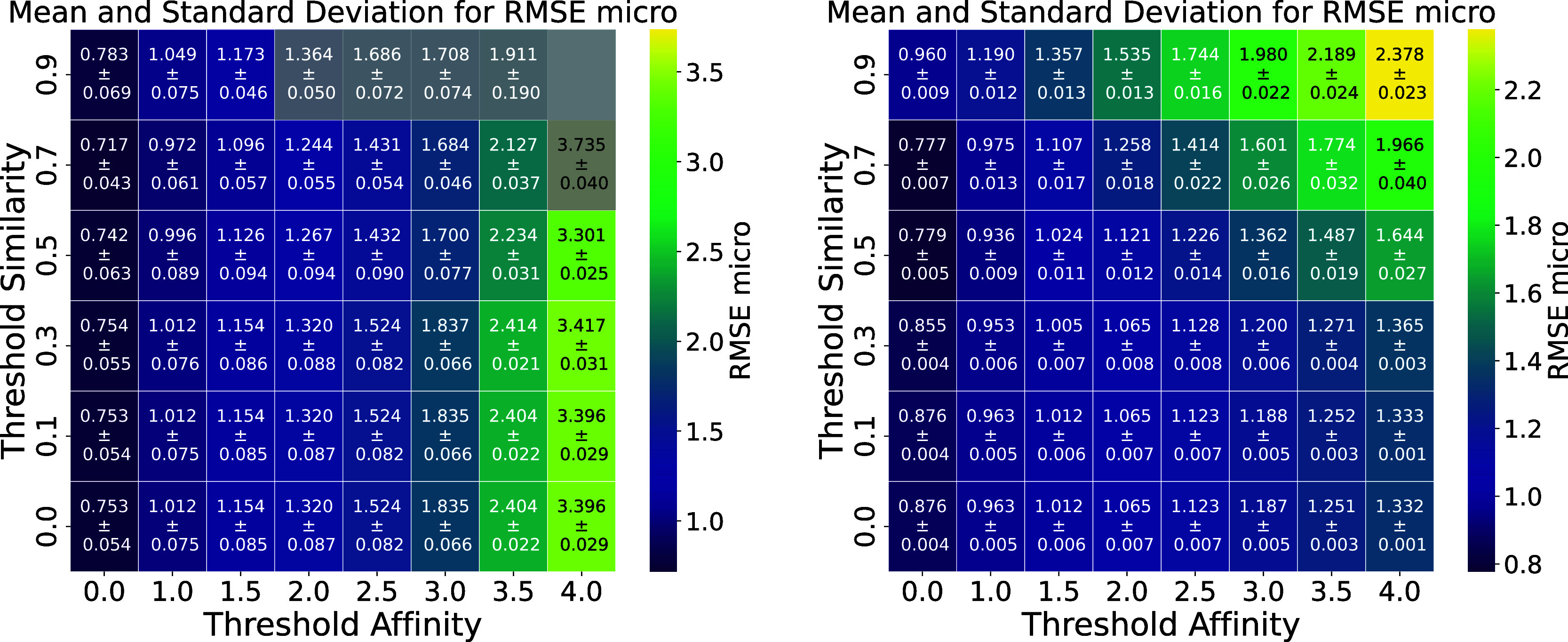
Heatmap of the RMSE_micro_ for the best DTI model trained from scratch for the KIBA (left) and BindingDB (right) data sets in the case of a compound-based splits, showing groups of compounds split by similarity and affinity thresholds. The values represent the mean ± standard deviation based on 3 experiments. The groups with fewer than 100 pairs are masked in gray. The model performs well in groups containing both ACs and non-ACs, particularly in the bottom left regions of the heatmaps. However, as the non-ACs are gradually filtered out and the model is faced with more difficult AC predictions, performance significantly declines, particularly in the upper right areas where thresholds for affinity and similarity are high.

For the KIBA data set, some upper-right groups lacked sufficient data, making these findings less robust. In contrast, the BindingDB data set has a higher number of compound pairs, leading to a more distinct pattern for the compound-based split (Figure 3, right). However, this improvement is not consistent across all targets, as shown in Supplementary Figure S4.

A similar pattern can be observed for the random split, as shown in Supplementary Figures S5 and S6. In both data sets, the random split demonstrates higher performance compared to the compound-based split, reflecting a setting where the training and test sets share a higher degree of similarity. While random splits are useful for evaluating model performance in scenarios with closely related data, the compound-based split represents a more challenging setting where the model must generalize to entirely novel compounds, making it more reflective in applications where unseen compounds are encountered.

### 3.3 Transfer Learning Settings

To enhance the prediction of affinities in novel compound–protein interactions, we employed transfer learning to leverage features learned from the AC task. This approach allows the DTI model to better handle the complexities of DTIs, improving its ability to generalize and deliver more accurate predictions, especially in challenging cases. For the KIBA data set, we explored three different configurations for transfer learning:

- Warm starting: this method involves fine-tuning, meaning that all the layers of the pretrained model are updated during training.
- Frozen weights: in this technique, the weights of the pretrained model are kept frozen and do not update during the training. Only the layers on top of the pretrained model are updated.
- Frozen weights with an additional layer: this approach is similar to the previous method of frozen weights, but with the addition of an extra layer in the drug branch before concatenation. This allows the frozen weights to be fine-tuned in the extra layer before the concatenation.

Among these configurations, warm starting achieved the best overall performance on the KIBA data set (see Table 2), so this approach was also applied for transfer learning on the BindingDB data set. The experiments involved transferring either only the drug encoder or both the drug and target encoders as shown in Supplementary Table S1. The idea is that the AC problem is largely compound-specific but is influenced by the context of the target protein. To account for this relationship, we explored the impact of incorporating a target-specific branch in the transfer learning framework. The parameters of the best DTI models for both data sets, across random and compound-based splits, are presented in Supplementary Tables S4 and S5 for the case of transferring only the drug encoder. For the case of transferring both drug and target encoders, the parameters are shown in Supplementary Tables S6 and S7, respectively. The heatmaps with models’ performance across different subgroups are shown in Supporting Information 9 and 10, resembling a pattern similar to that of the baseline DTI models. The effect of transfer learning is evaluated using differential heatmaps, which are discussed in the next section.

**Table 2:** Overall Performance of the DTI Models

**task**	**data set (split)**	**TL** ^a^ **setting**	**transferred encoder(s)**	RMSE_micro_ ^b^
DTI (baseline)	KIBA (random)			0.447 ± 0.007
	KIBA (compound-based)			0.669 ± 0.046
	BindingDB (random)			0.787 ± 0.008
	BindingDB (compound-based)			0.933 ± 0.004
DTI (TL)	KIBA (random)	warm starting	drug	0.443 ± 0.009
		freezing weights		0.485 ± 0.004
		freezing weights + layer		0.447 ± 0.012
		warm starting	drug + target	0.439 ± 0.010
		freezing weights		0.492 ± 0.006
		freezing weights + layer		0.445 ± 0.006
	KIBA (compound-based)	warm starting	drug	0.642 ± 0.002
		freezing weights		0.719 ± 0.012
		freezing weights + layer		0.675 ± 0.002
		warm starting	drug + target	0.612 ± 0.004
		freezing weights		0.739 ± 0.017
		freezing weights + layer		0.692 ± 0.017
	BindingDB (random)	warm starting	drug	0.808 ± 0.007
		warm starting	drug + target	0.765 ± 0.001
	BindingDB (compound-based)	warm starting	drug	0.938 ± 0.010
		warm starting	drug + target	0.899 ± 0.003

a TL: transfer learning.

b The values represent the mean ± standard deviation based on 3 experiments.

The figure in the graphical abstract presents an example of ACs in the context of affinity (*K*_i_) prediction in the case of the compound-based split of the BindingDB data set. The figure highlights two structurally similar compounds targeting the apoptosis regulator Bcl-2, with key structural differences marked in red. Despite their similarity, these compounds exhibit significantly different binding affinities, with log-scale *K*_i_ values of 9.79 and 7.29 nM, illustrating the pronounced activity changes typical of ACs. For the left compound, both the initial and transfer learning-based predictions are accurate. However, for the right compound, the predictions made without transfer learning significantly deviate from the actual *K*_i_ value. In contrast, the predictions that incorporate transfer learning (involved both drug and target encoders) closely align with the true affinity. This suggests that the model has started to effectively distinguish between the drugs.

### 3.4 Evaluation of the Transfer Learning Effect

Differential heatmaps were created to evaluate the impact of transfer learning by comparing the best baseline model, trained from scratch, with the best transfer learning model on the test set. The evaluation process involves subtracting the RMSE values of the transfer learning model from those of the baseline model within each subgroup. In the context of RMSE, where lower values indicate better performance, a positive difference in the heatmap suggests that the transfer learning model outperforms the baseline by achieving lower RMSE values, thus providing more accurate predictions.

For example, to evaluate the effect of transferring both the drug and target encoders on the KIBA data set in case of the compound-based split and warm starting setting, the RMSE values from the transfer learning model (Supplementary Figure S18) were subtracted from those of the baseline model (Figure 3). This calculation produced the differential heatmap presented in Figure 4, left. The same approach was used to generate all differential heatmaps.

**Figure 4:**
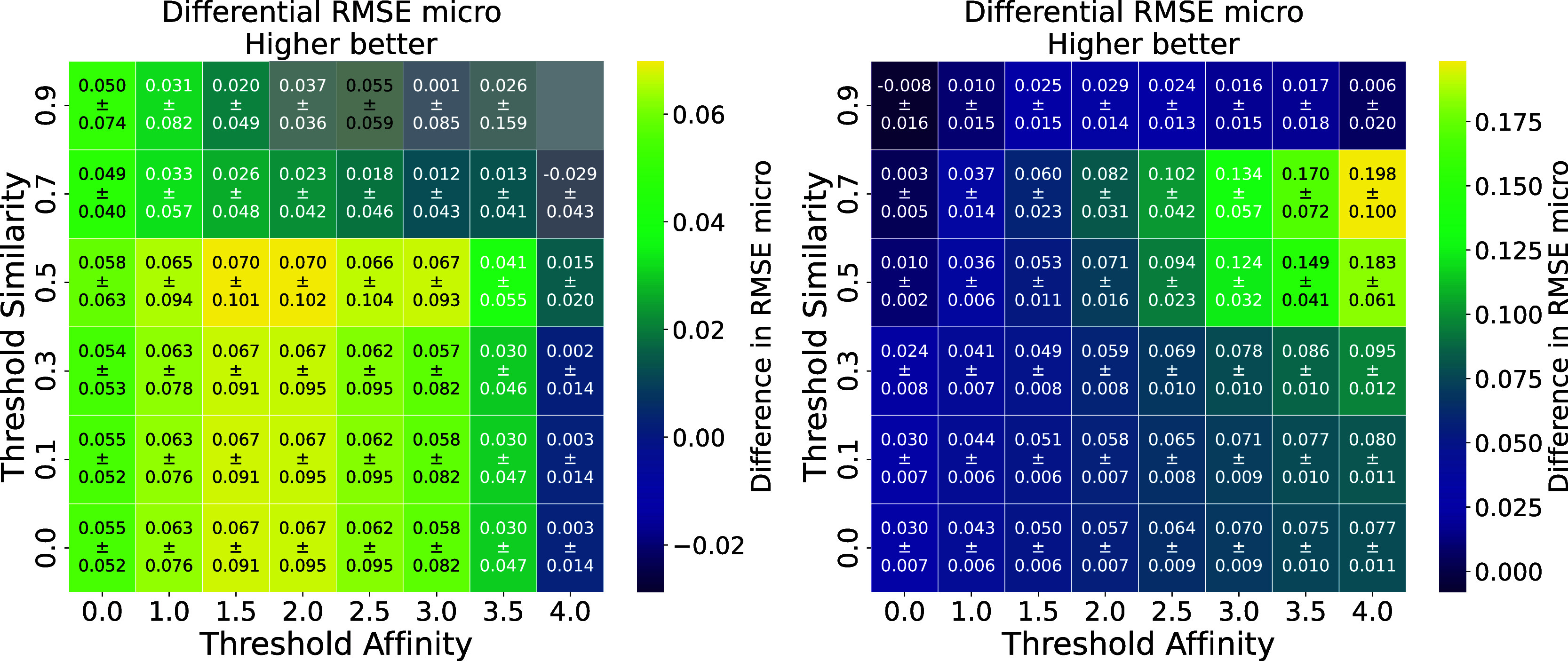
Differential heatmap of the RMSE_micro_ for the best DTI model­(transfer learning involving both drug and target encoders, warm start) for the KIBA (left) and BindingDB (right) data sets in the case of a compound-based splits, showing groups of compounds split by similarity and affinity thresholds. Transfer learning models outperform baseline models at lower affinity thresholds (left column), as indicated by positive RMSE differences. However, negative RMSE values at higher thresholds (top right) suggest poorer performance in cases with very similar compounds and large affinity variations. This indicates that transfer learning works well for simpler cases but struggles with more challenging ACs.

In the case of the KIBA data set, for both random and compound-based splits, transfer learning with the warm starting setting consistently outperformed the baseline model, whereas models using frozen weights (both with and without an additional layer) did not show improved performance. This highlights the importance of fine-tuning all layers of the pretrained model to effectively adapt the learned features for the DTI task.

Notably, transferring only the drug encoder resulted in worse performance across all settings and splits. This outcome is unsurprising, as the AC task, while primarily compound-specific, inherently relies on the contextual information provided by the target protein. Without the target encoder, the transfer learning model lacks the critical context needed to effectively generalize to the DTI task. These findings underscore the anticipated importance of incorporating both drug and target encoders in transfer learning approaches for DTI tasks, aligning with the understanding that drug-target interactions are inherently dependent on the interplay between compound structure and protein context.

In the compound-based split of the KIBA data set (Figure 4, left), transfer learning with warm starting setting and both the drug and target encoders transferred shows improvements at lower affinity thresholds, as indicated by positive RMSE_micro_ differences in the leftmost columns, demonstrating effectiveness in simpler cases. However, performance declines at higher affinity and similarity thresholds (upper right), reflecting challenges in handling extreme AC cases. Furthermore, the standard deviations are often comparable to or larger than the mean, highlighting variability in the model’s predictions and reduced stability.

In the case of random split but with the same warm starting setting and transfer of both encoders, the results are slightly better for the KIBA data set (Supplementary Figure S31) compared to the compound-based split. This indicates that the random split provides a less stringent evaluation, as the overlap of compounds between training and testing sets allows the model to encounter familiar compounds, potentially resulting in data leakage and inflated performance metrics. In contrast, with the compound-based split, where the same compounds are consistently used across both AC and DTI tasks, we observed a performance drop. While this drop is expected, it provides a more realistic evaluation of the model’s generalization abilities, offering a more accurate measure of how the model will perform on new, unseen data.

However, one major challenge when generalizing to new drugs and proteins is that the data set sizes significantly diminish. As we apply various rules and constraints to ensure proper generalization, the data set becomes much smaller, which can impact the robustness of the models and their ability to make accurate predictions. In some cases, particularly for extreme AC instances, the limited number of samples makes it difficult to draw meaningful conclusions, as the small sample size is not representative. On the other hand, having a large data set is advantageous, as it provides more robust data for analysis compared to a smaller data set focused on a single protein.^17^

The compound-based split of the BindingDB data set (Figure 4, right) presents a more favorable trend, with transfer learning showing consistent improvements across most regions. Positive RMSE_micro_ differences are observed even at moderate and higher affinity thresholds, suggesting that the larger data set size of BindingDB provides better support for generalization in complex scenarios. Nonetheless, similar to KIBA, performance gains are reduced at the highest thresholds of similarity, indicating that extreme AC cases remain challenging. While the standard deviations in the BindingDB data set are generally smaller and more controlled compared to those in the KIBA data set, particularly at higher thresholds, they are still relatively large in some regions. This is especially noticeable in groups with high similarity and affinity thresholds, where variability remains significant. These observations highlight that, although the larger data set size of BindingDB reduces variability and enhances stability in most regions, challenging subgroups with extreme thresholds still present inconsistencies in the model’s predictive performance.

However, for the BindingDB data set (Supplementary Figure S36), the results are slightly worse under the random split compared to the compound-based split. This suggests that the larger and more diverse nature of the BindingDB data set may expose limitations in the model’s generalization when evaluated in a random split scenario.

The RMSE_macro_ values for both data sets are in the case of both encoders transfer learning and warm starting setting is illustrated in Supplementary Figure S37. Positive values of differences in RMSE_macro_ are dispersed across the heatmap but are generally lower in magnitude compared to the microaveraging heatmap. This indicates that while the transfer learning model shows improvement in certain areas, these improvements are less consistent across different groups. This inconsistency may be attributed to the nature of the proteins or variations in the amount of affinity data available for different targets.

### 3.5 Further Implications

Prior work in DTI prediction has often overlooked the impact of ACs or treated AC prediction as a separate challenge, focusing primarily on molecular similarity-based approaches. These methods, while effective in many cases, struggle to account for the complex, discontinuous structure–activity relationships introduced by AC phenomena. This work stands apart by directly integrating AC awareness into DTI prediction through transfer learning. By leveraging knowledge from AC prediction tasks, our approach not only addresses these challenges but also improves the generalizability and accuracy of predictive models, particularly in scenarios involving structurally similar yet functionally distinct compounds.

Several state-of-the-art models have been developed for drug–target interaction prediction on benchmark data sets such as KIBA and BindingDB. Notable examples include DeepDTA,^31^ which employs convolutional neural networks on SMILES and protein sequences; GraphDTA,^37^ which leverages graph neural networks to represent molecular structures; TransformerCPI,^38^ which applies transformer-based architectures for capturing long-range dependencies; and SSnet,^39^ which processes fingerprints with fully connected layers and the protein’s secondary structure information with a convolutional network. While these models represent important advances on the DTI task, they do not consider the concept of AC or try to incorporate it via transfer learning, which is the focus of the current study. Our work complements these efforts in two ways. First, we demonstrate that a two-branch architecture, conceptually similar to the state-of-the-art methods, struggles to predict the affinity of AC examples. Second, we explore how AC-informed transfer learning can improve DTI prediction, particularly in challenging cases where molecular similarity assumptions no longer hold. Future research could explore integrating AC transfer learning with other architectures such as CNNs, GNNs, and transformers, to improve the affinity prediction performance of AC molecules.

Moreover, the incorporation of both drug and target encoders in transfer learning highlights the interdependent nature of these two elements in accurately modeling DTIs. By illustrating the limitations of transferring only the drug encoder, our study provides theoretical support for the development of multifaceted models that leverage contextual target information. This insight aligns with the fact that molecular similarity alone is insufficient for predicting biological interactions, emphasizing the importance of incorporating protein-specific context in predictive models.

Furthermore, the development of a universal model for AC prediction across all targets simplifies the modeling process. This approach reduces the need for target-specific models, which often require substantial computational and data resources. This study underscores the importance of further exploring the role of transfer learning in handling data scarcity and imbalance, which are common challenges in biomedical data sets. Future research could build on our findings by investigating additional transfer learning configurations, such as domain-specific pretraining or incorporating structural protein information into target encoders. Additionally, the variability observed in the performance of transfer learning models across data sets and splits suggests that further work is needed to tailor these approaches to the specific characteristics of data sets.

One limitation of this work arises from the use of integrated bioactivity scores in the KIBA data set for AC classification. While the KIBA score effectively merges multiple bioactivity end points such as *K*_i_, *K*_d_, and IC50 for general DTI prediction, this integration introduces a degree of variability that may not be ideal for detecting ACs. As such, the use of mixed end points could partly explain the observed inconsistencies in AC-related results. Future studies could address this by employing data sets with uniform and directly comparable end points (e.g., *K*_i_-only) to improve the robustness and interpretability of AC prediction outcomes.

## 4 Conclusions

In this work, we investigated whether transfer learning techniques from AC prediction tasks can improve the overall performance of the DTI prediction model. First, we developed AC models to predict ACs, with the BindingDB data set yielding better performance than KIBA, likely due to its larger size. The AC models provided a universal framework for identifying ACs without the need for separate models per target. Next, we created DTI baseline models and confirmed that predicting DTI in the context of ACs remains a significant challenge, as models struggled with AC pairs. To address this, we applied a transfer learning approach by incorporating knowledge from the AC prediction task into the DTI model. This involved three strategies: warm starting, freezing weights, and freezing weights with an additional layer. Differential heatmaps were used to assess the performance of the transfer learning models against the baseline models.

For both the KIBA and BindingDB data sets, transfer learning models with warm starting showed consistent improvements over the baseline, particularly in scenarios involving high similarity thresholds and varying affinity differences. In contrast, the models using frozen weights, either with or without an additional layer, generally performed the same or worse than the baseline models, failing to provide notable improvements. Moreover, when comparing the transfer of only the drug encoder versus both the drug and target encoders, transferring both encoders yielded superior results. This was especially evident in the case of high similarity thresholds, where the full transfer led to better generalization. However, some regions still exhibited less consistent gains, particularly in the KIBA data set, where certain subgroups showed comparable or slightly worse performance. This variability underscores the dependency of transfer learning effectiveness on the specific characteristics of the data, such as the nature of the targets and the availability of affinity data.

Overall, our findings demonstrate that transfer learning from AC prediction tasks can significantly improve DTI model performance, particularly in predicting ACs. This approach contributes to more accurate and robust drug-target interaction predictions, with promising implications for real-world drug discovery and development.

## Supplementary Material



## Data Availability

The code of this study was developed using PyTorch Lightning module and is accessible via the GitHub repository https://github.com/reginaib/AC-DTI. This repository also includes the scripts for data preprocessing and postprocessing, as well as the data sets and pretrained models, ensuring the reproducibility of the results.

## Notes

(ZIP)
Overview of experimental setup; details on hyperparameter ranges; hyper-parameters and performance of best AC models; hidden state visualization; hyper-parameters and performance of best DTI models; number of pairs heatmaps; performance of the baseline models with compound-based and random splits; and performance of the models when transferring only the drug encoder and both drug and target encoders for both KIBA and BidingDB data sets (PDF)
