# Supplementary material for: Enhancing Drug-Target Interaction Prediction through Transfer Learning from Activity Cliff Prediction Tasks
Source: J Chem Inf Model. 2025 Jun 30;65(13):6558–67. doi: 10.1021/acs.jcim.5c00484 (PMC12264954; doi:10.1021/acs.jcim.5c00484)
Supplement: Supplementary file 1 [file ci5c00484_si_002.pdf]

Enhancing Drug-Target Interaction Prediction through Transfer Learning from Activity Cliff Prediction Tasks

Regina Ibragimova, Dimitrios Iliadis, and Willem Waegeman*

Department of Data Analysis and Mathematical Modelling, Ghent University, Coupure Links, Ghent, 9000, Belgium

E-mail: willem.waegeman@ugent.be

Number of pages: 27

Number of figures: 37

Number of tables: 7

1 Overview of experimental setup

This section provides an overview of the experimental setup utilized in this study, as summarized in Table **S1**. The table outlines the tasks performed, datasets used (including their splitting strategies), transfer learning settings, transferred components (drug and/or target encoders), and links to the corresponding experiments on the Weights and Biases platform.

For the AC task, models were trained separately for two datasets (KIBA and BindingDB) using random and compound-based splits. For the DTI task, both baseline models (trained from scratch) and transfer learning models were evaluated on the same datasets. Transfer

learning involved three strategies—warm starting, freezing weights, and freezing weights with an additional layer—applied to either the drug encoder alone or both drug and target encoders. This setup ensured a comprehensive evaluation of the models under various conditions, providing insights into the performance of baseline and transfer learning approaches across different datasets and experimental settings.

Table S1: Overview of experimental setup with Weights and Biases projects' links.

Task	Dataset (Split)	TL[1] Setting	Transferred Encoder(s)	Links
AC	KIBA (Random)	-	-	HPO[2], Best[3]
	KIBA (Compound-based)	-	-	HPO, Best
	BindingDB (Random)	-	-	HPO, Best
	BindingDB (Compound-based)	-	-	HPO, Best
DTI (Baseline)	KIBA (Random)	-	-	HPO, Best
	KIBA (Compound-based)	-	-	HPO, Best
	BindingDB (Random)	-	-	HPO, Best
	BindingDB (Compound-based)	-	-	HPO, Best
DTI (TL)	KIBA (Random)	Warm Starting	Drug	HPO, Best
		Freezing Weights		HPO, Best
		Freezing Weights + Layer		HPO, Best
		Warm Starting	Drug + Target	HPO, Best
		Freezing Weights		HPO, Best
		Freezing Weights + Layer		HPO, Best
	KIBA (Compound-based)	Warm Starting	Drug	HPO, Best
		Freezing Weights		HPO, Best
		Freezing Weights + Layer		HPO, Best
		Warm Starting	Drug + Target	HPO, Best
		Freezing Weights		HPO, Best
		Freezing Weights + Layer		HPO, Best
	BindingDB (Random)	Warm Starting	Drug	HPO, Best
		Warm Starting	Drug + Target	HPO, Best
	BindingDB (Compound-based)	Warm Starting	Drug	HPO, Best
		Warm Starting	Drug + Target	HPO, Best

[a]TL: Transfer Learning.
[b]HPO: Hyper-parameter optimization.
[c]Best: Training of the best model.

2 Hyper-parameter ranges

In the following section, we provide the hyper-parameter space we explore in both AC and DTI tasks:

- number of hidden layers in drug encoder: [1, 2, 3, 4]
- drug hidden layer size: [32, 64, 128, 256, 512, 768, 1024]
- target embedding size: [32, 64, 128, 256, 512, 768, 1024]
- head hidden layer size: [32, 64, 128, 256, 512, 768, 1024]
- learning rate: [0.00001, 0.00003, 0.0001, 0.0003, 0.001]
- dropout rate: [0.01, 0.1, 0.2, 0.3, 0.4, 0.5]

In the transfer learning setting involving freezing weights and adding an extra layer, the following hyper-parameters were explored:

- drug additional hidden layer size: [32, 64, 128, 256, 512, 768, 1024]
- dropout rate: [0.01, 0.1, 0.2, 0.3, 0.4, 0.5]

3 Hyper-parameters and performance of best AC models

Table S2: Combinations of hyper-parameters of best AC models on KIBA and BindindDB datasets

	Datasets (random split)		Datasets (compound-based split)	
Hyper-parameters	KIBA	BindingDB	KIBA	BindingDB
# Hidden Layers in Drug Encoder	1	4	2	1
Drug Hidden Layer Size	1024	1024	128	1024
Target Embedding Size	768	512	256	512
Head Hidden Layer Size	768	1024	768	1024
Learning Rate	0.0003	0.0003	0.001	0.001
Dropout Rate	0.01	0.2	0.2	0.4

Table S3: Performance of best AC models on KIBA and BindindDB datasets

	Random split		Compound-based split	
Metric	KIBA	BindingDB	KIBA	BindingDB
Precision	0.627	0.664	0.338	0.385
Recall	0.644	0.856	0.386	0.622
F1-score	0.636	0.748	0.360	0.476
MCC	0.583	0.641	0.293	0.235
Balanced Accuracy	0.795	0.843	0.655	0.631

4 Hidden state visualization

To better understand the model's ability to discriminate between ACs and non-ACs, the input and the hidden states after the first and fourth layers of the drug encoder in the AC model trained on the BindingDB dataset (random split) were visualized using Uniform Manifold Approximation and Projection (UMAP). For a specific protein (tissue-type plasminogen activator), all pairs involving the same compound (referred to as the 'Reference Compound' and colored yellow in the plot) were extracted and plotted. The other compounds are colored based on their pairing with the reference compound: those in AC cliff pairs are colored teal, while non-AC pairs are colored purple. As seen in Supplementary Figure **S1**, the distinction is not immediately present in the feature space. However, as the representations pass through layers, the model increasingly succeeds in

discriminating the points, resulting in clearer clusters. Additionally, it can be observed that the reference compound is closer to non-ACs, while ACs are further away, indicating that the model is able to effectively distinguish ACs.

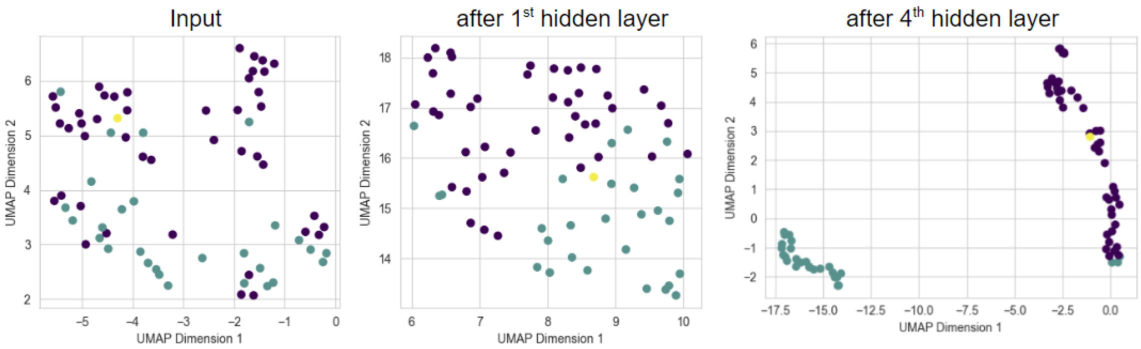

Figure S1: UMAP of input features and hidden states of the AC model's drug encoder (on the BindingDB dataset, random split).

5 Hyper-parameters and performance of best DTI models

Table S4: The parameters of the best models obtained using transfer learning (random split datasets, transferring only drug encoder)

	KIBA				BindingDB	
Projectile	Baseline	TL warm starting	TL FW	TL FW + Layer	Baseline	TL warm starting
# Hidden Layers in Drug Encoder	1	1	1	1	1	4
Drug Hidden Layer Size	256	1024	1024	1024	1024	1024
Target Embedding Size	256	128	256	256	32	256
Head Hidden Layer Size	1024	768	1024	1024	768	1024
Learning Rate	0.0001	0.001	0.0003	0.0003	0.0003	0.0001
Dropout Rate	0.01	0.2	0.1	0.1	0.1	0.3
Additional layer size	-	-	-	1024	-	-
Additional layer dropout	-	-	-	0.5	-	-

Table S5: The parameters of the best models obtained using transfer learning (compound-based split datasets, transferring only drug encoder)

	KIBA				BindingDB	
Projectile	Baseline	TL[1] warm starting	TL FW[1]	TL FW + Layer	Baseline	TL warm starting
# Hidden Layers in Drug Encoder	1	2	2	2	1	1
Drug Hidden Layer Size	768	128	128	128	768	1024
Target Embedding Size	768	128	64	64	512	512
Head Hidden Layer Size	512	1024	1024	1024	1024	256
Learning Rate	0.001	0.0001	0.0001	0.0001	0.00003	0.00003
Dropout Rate	0.1	0.2	0.1	0.1	0.5	0.2
Additional layer size	-	-	-	1024	-	-
Additional layer dropout	-	-	-	0.4	-	-

TL: Transfer Learning, FW: Freezing Weights.
TL: Transfer Learning, FW: Freezing Weights.

Table S6: The parameters of the best models obtained using transfer learning (random split datasets, transferring both drug and target encoders)

	KIBA				BindingDB	
Projectile	Baseline	TL warm starting	TL FW	TL FW + Layer	Baseline	TL warm starting
# Hidden Layers in Drug Encoder	1	1	1	1	1	1
Drug Hidden Layer Size	256	1024	1024	1024	1024	1024
Target Embedding Size	256	768	768	768	32	512
Head Hidden Layer Size	1024	1024	1024	1024	768	1024
Learning Rate	0.0001	0.0003	0.0001	0.0001	0.0003	0.00003
Dropout Rate	0.01	0.2	0.1	0.1	0.1	0.3
Additional layer size	-	-	-	1024	-	-
Additional layer dropout	-	-	-	0.01	-	-

Table S7: The parameters of the best models obtained using transfer learning (compound-based split datasets, transferring both drug and target encoders)

	KIBA				BindingDB	
Projectile	Baseline	TL warm starting	TL FW	TL FW + Layer	Baseline	TL warm starting
# Hidden Layers in Drug Encoder	1	2	2	2	1	1
Drug Hidden Layer Size	768	128	128	128	768	1024
Target Embedding Size	768	256	256	256	512	512
Head Hidden Layer Size	512	1024	768	768	1024	768
Learning Rate	0.001	0.0003	0.0003	0.0003	0.00003	0.0001
Dropout Rate	0.1	0.1	0.2	0.2	0.5	0.2
Additional layer size	-	-	-	1024	-	-
Additional layer dropout	-	-	-	0.2	-	-

TL: Transfer Learning, FW: Freezing Weights.

TL: Transfer Learning, FW: Freezing Weights.

6 Number of pairs heatmaps

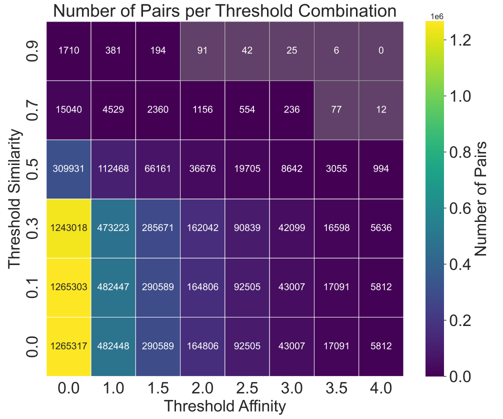

(a)

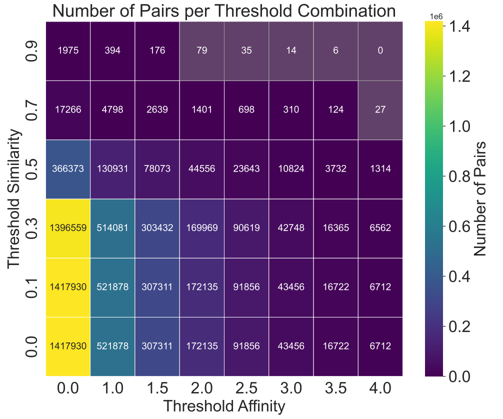

(b)

Figure S2: Number of pairs per groups in test sets in KIBA random (a) and compound-based (b) splits. It was found that not all the subgroups had a sufficient number of pairs, so those below 100 pairs were masked by grey color. The numbers are shown for pairs of compounds targeting the same protein, meaning that targets with an affinity for only one drug were excluded.

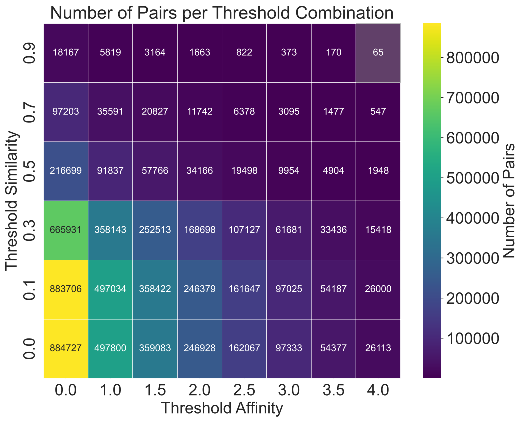

(a)

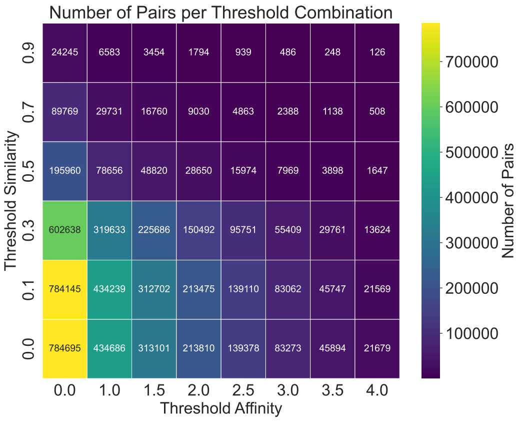

(b)

Figure S3: Number of pairs per groups in test sets in BindingDB random (a) and compound-based (b) splits. Groups with less than 100 pairs were masked by grey color.

7 Performance of the baseline models with compound-based split

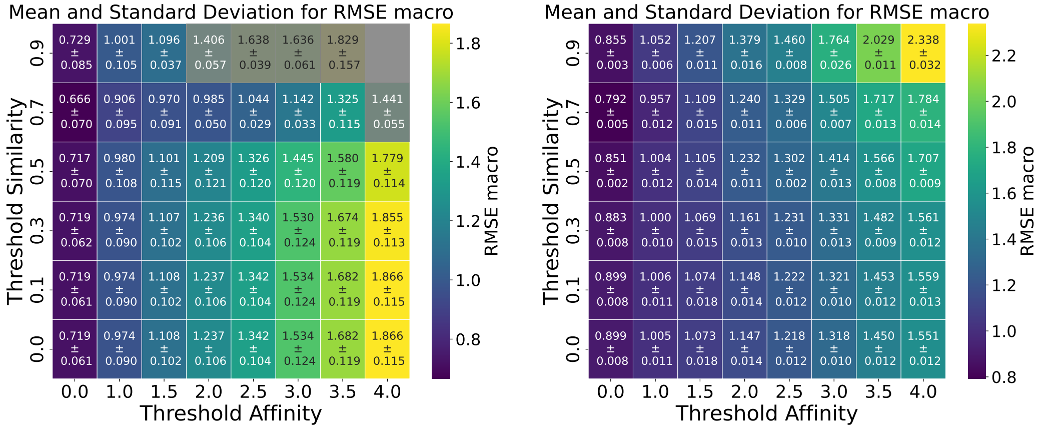

Figure S4: The heatmap of the $RMSE_{macro}$ for the best DTI model trained from scratch for the KIBA (left) and BindingDB (right) datasets in the case of a compound-based splits, showing groups of compounds split by similarity and affinity thresholds. The values represent the mean ± standard deviation based on 3 experiments. The groups with fewer than 100 pairs are masked in gray.

8 Performance of the baseline models with random split

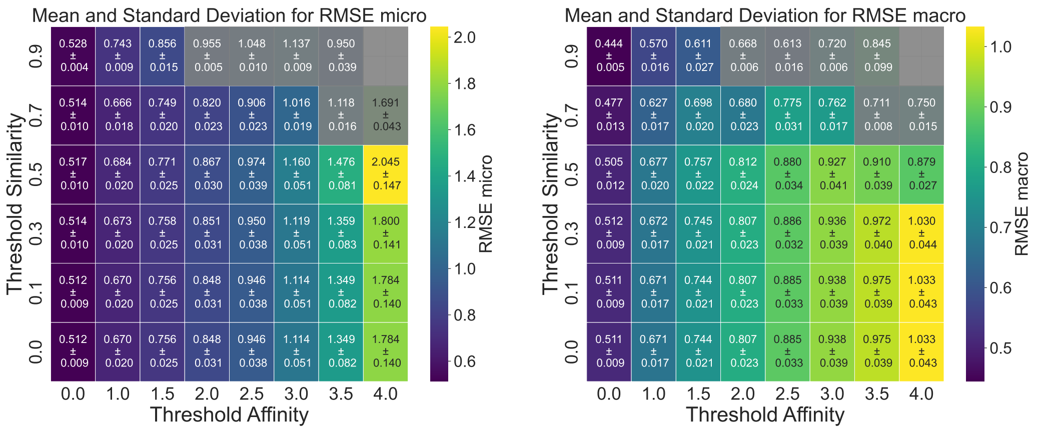

Figure S5: The heatmap of the $RMSE_{micro}$ (left) and $RMSE_{macro}$ (right) for the best DTI model trained from scratch, showing groups of compounds split by similarity and affinity thresholds for the KIBA dataset in the case of a random split. The values represent the mean ± standard deviation based on 3 experiments. The groups with fewer than 100 pairs are masked in gray.

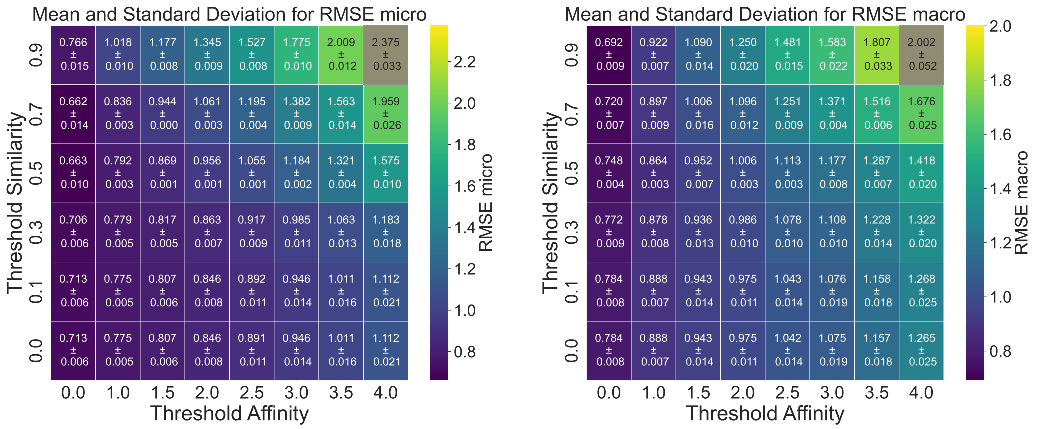

Figure S6: The heatmap of the $RMSE_{micro}$ (left) and $RMSE_{macro}$ (right) for the best DTI model trained from scratch, showing groups of compounds split by similarity and affinity thresholds for the BindingDB dataset in the case of a random split. The values represent the mean ± standard deviation based on 3 experiments. The groups with fewer than 100 pairs are masked in gray.

9 Performance of the models when transferring only the drug encoder

9.1 KIBA dataset

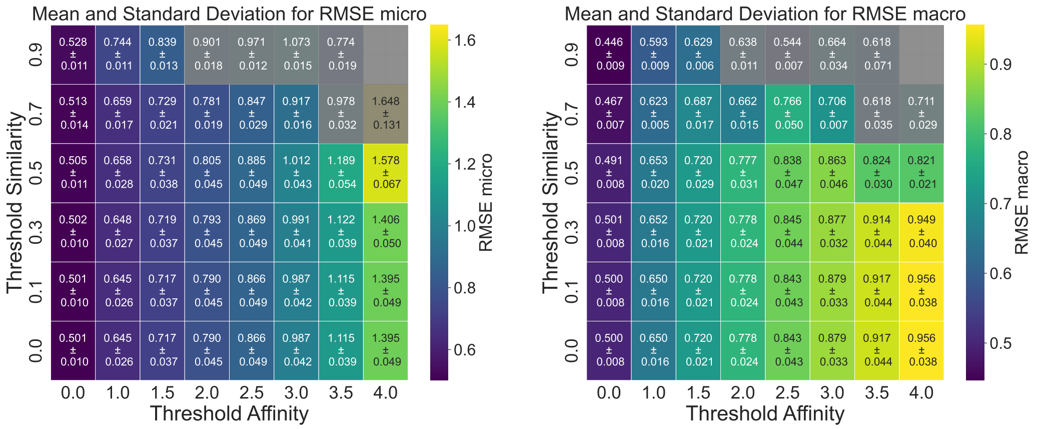

Figure S7: The heatmap of the $RMSE_{micro}$ (left) and $RMSE_{macro}$ (right) of the best DTI model (transfer learning involving only the drug encoder, **warm start**) for groups of compounds split by similarity and affinity thresholds for KIBA (random split).

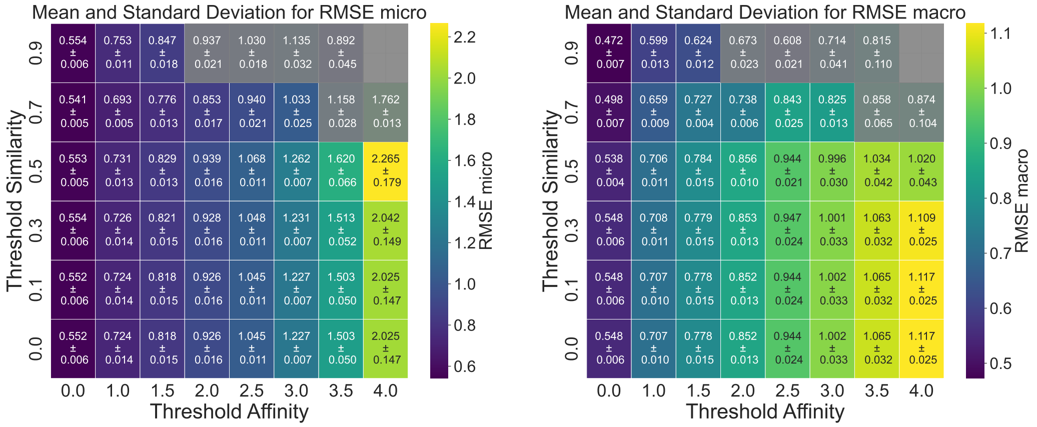

Figure S8: The heatmap of the $RMSE_{micro}$ (left) and $RMSE_{macro}$ (right) of the best DTI model (transfer learning involving only the drug encoder, **with freezing weights**) for groups of compounds split by similarity and affinity thresholds for KIBA (random split).

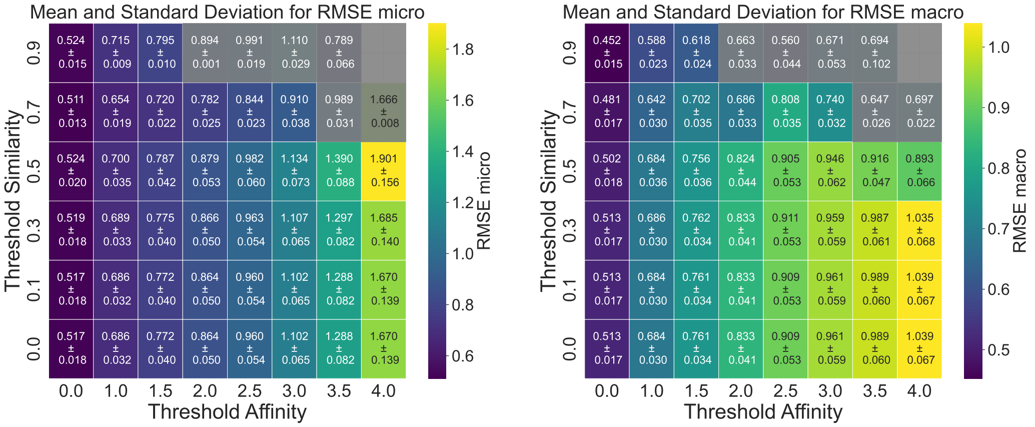

Figure S9: The heatmap of the $RMSE_{micro}$ (left) and $RMSE_{macro}$ (right) of the best DTI model (transfer learning involving only the drug encoder, **with freezing weights and adding an extra layer**) for groups of compounds split by similarity and affinity thresholds for KIBA (random split split).

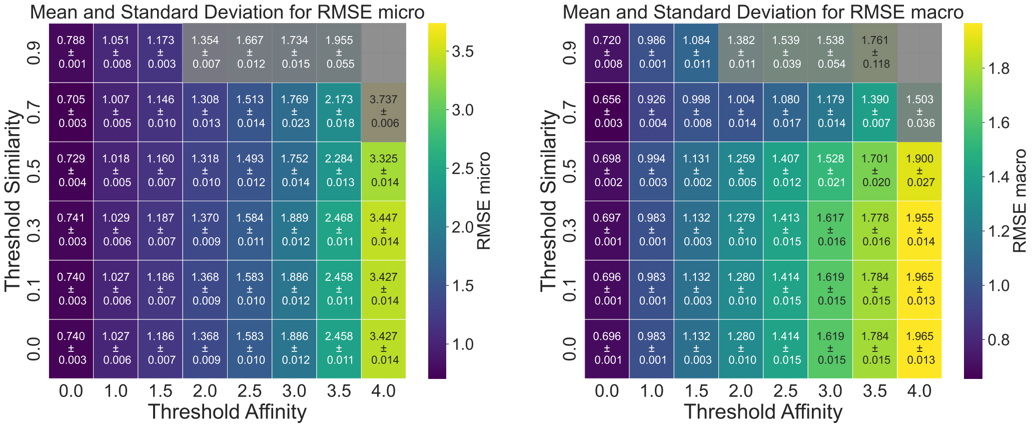

Figure S10: The heatmap of the $RMSE_{micro}$ (left) and $RMSE_{macro}$ (right) of the best DTI model (transfer learning involving only the drug encoder, **warm start**) for groups of compounds split by similarity and affinity thresholds for KIBA (compound-based split).

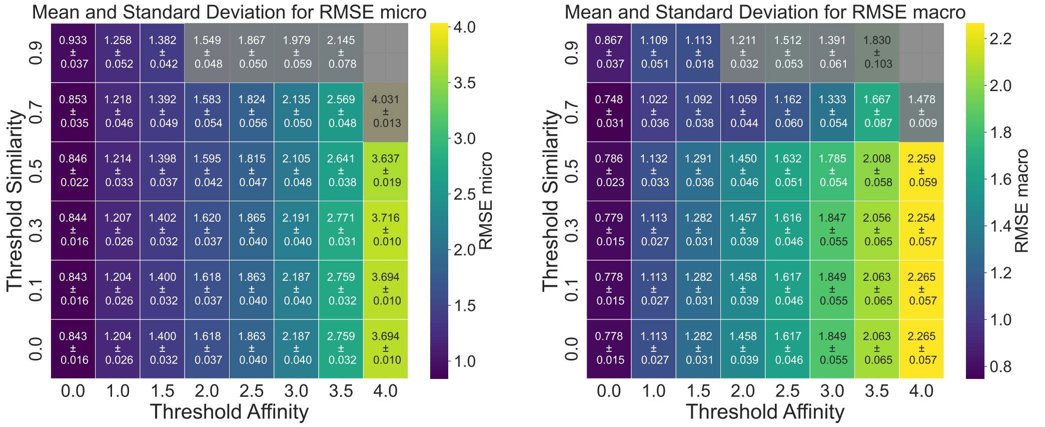

Figure S11: The heatmap of the $RMSE_{micro}$ (left) and $RMSE_{macro}$ (right) of the best DTI model (transfer learning involving only the drug encoder, **with freezing weights**) for groups of compounds split by similarity and affinity thresholds for KIBA (compound-based split).

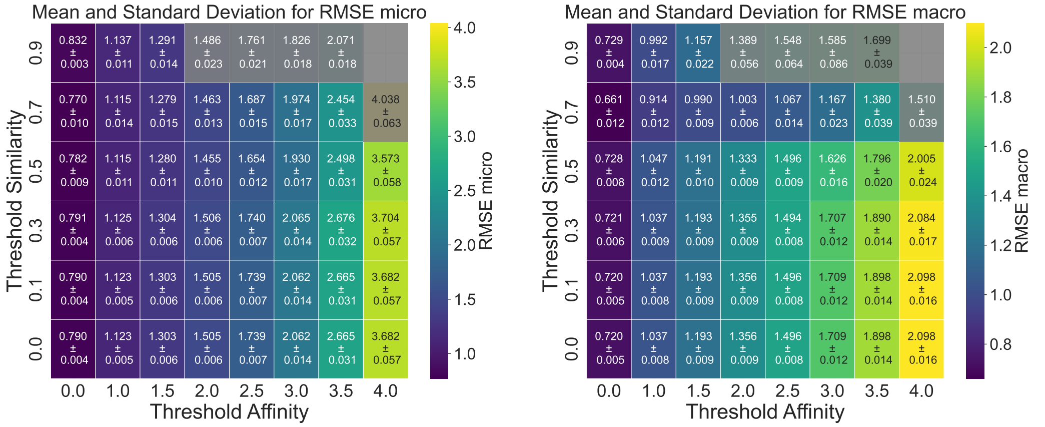

Figure S12: The heatmap of the $RMSE_{micro}$ (left) and $RMSE_{macro}$ (right) of the best DTI model (transfer learning involving only the drug encoder, **with freezing weights and adding an extra layer**) for groups of compounds split by similarity and affinity thresholds for KIBA (compound-based split).

9.2 BindingDB dataset

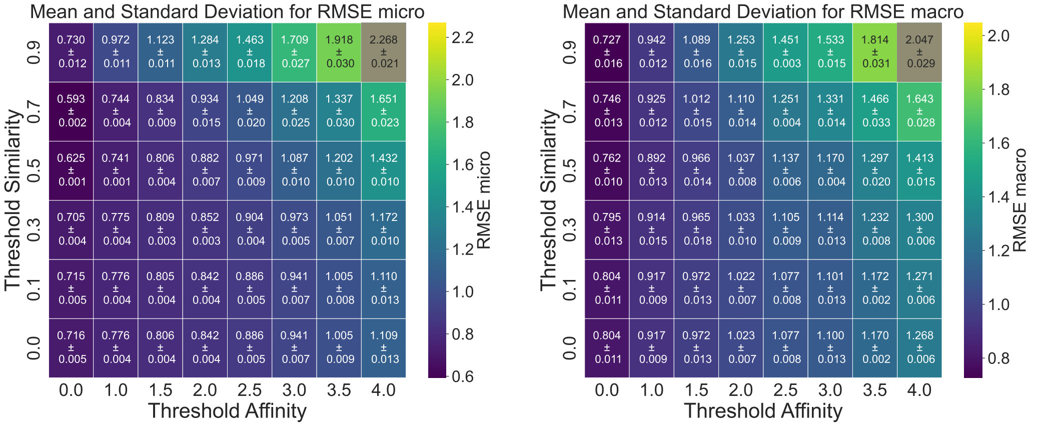

Figure S13: The heatmap of the $RMSE_{micro}$ (left) and $RMSE_{macro}$ (right) of the best DTI model (transfer learning involving only the drug encoder, **warm start**) for groups of compounds split by similarity and affinity thresholds for BindingDB (random split).

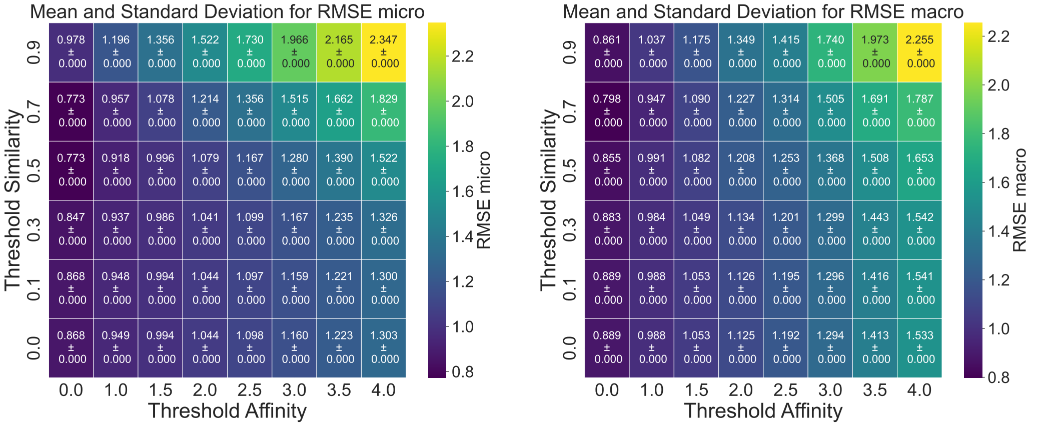

Figure S14: The heatmap of the $RMSE_{micro}$ (left) and $RMSE_{macro}$ (right) of the best DTI model (transfer learning involving only the drug encoder, **warm start**) for groups of compounds split by similarity and affinity thresholds for BindingDB (compound-based split).

10 Performance of the models when transferring both drug and target encoders

10.1 KIBA dataset

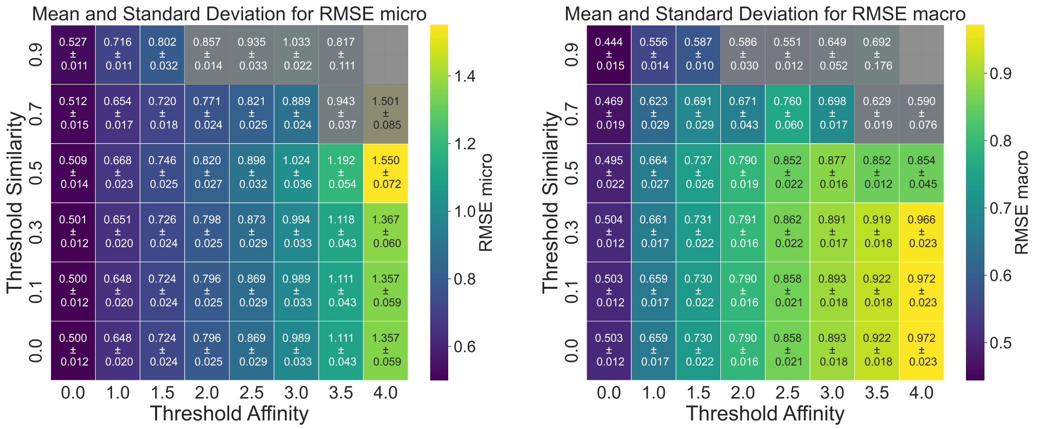

Figure S15: The heatmap of the $RMSE_{micro}$ (left) and $RMSE_{macro}$ (right) of the best DTI model (transfer learning involving both drug and target encoders, **warm start**) for groups of compounds split by similarity and affinity thresholds for KIBA (random split).

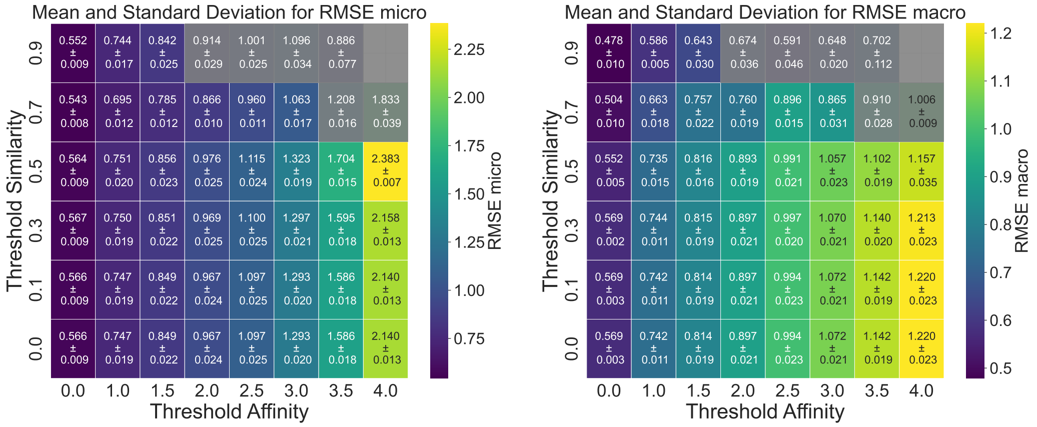

Figure S16: The heatmap of the $RMSE_{micro}$ (left) and $RMSE_{macro}$ (right) of the best DTI model (transfer learning involving both drug and target encoders, **with freezing weights**) for groups of compounds split by similarity and affinity thresholds for KIBA (random split).

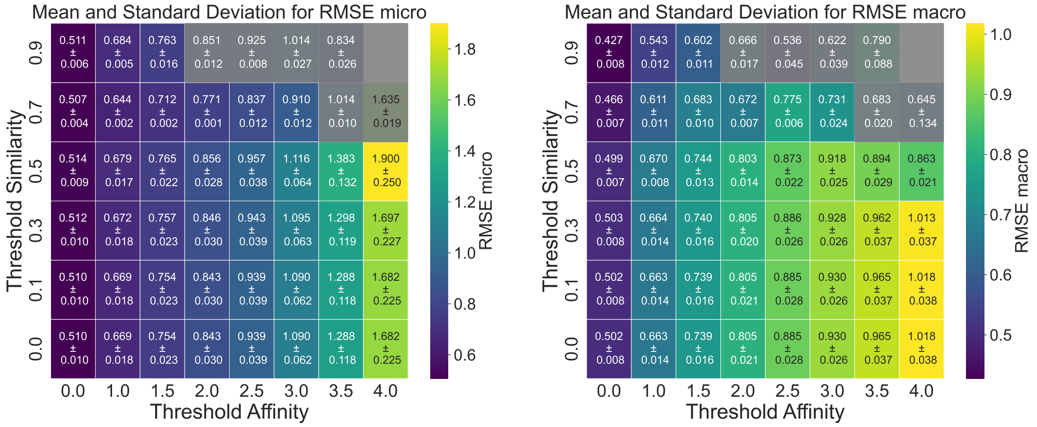

Figure S17: The heatmap of the $RMSE_{micro}$ (left) and $RMSE_{macro}$ (right) of the best DTI model (transfer learning involving both drug and target encoders, **with freezing weights and adding an extra layer**) for groups of compounds split by similarity and affinity thresholds for KIBA (random split split).

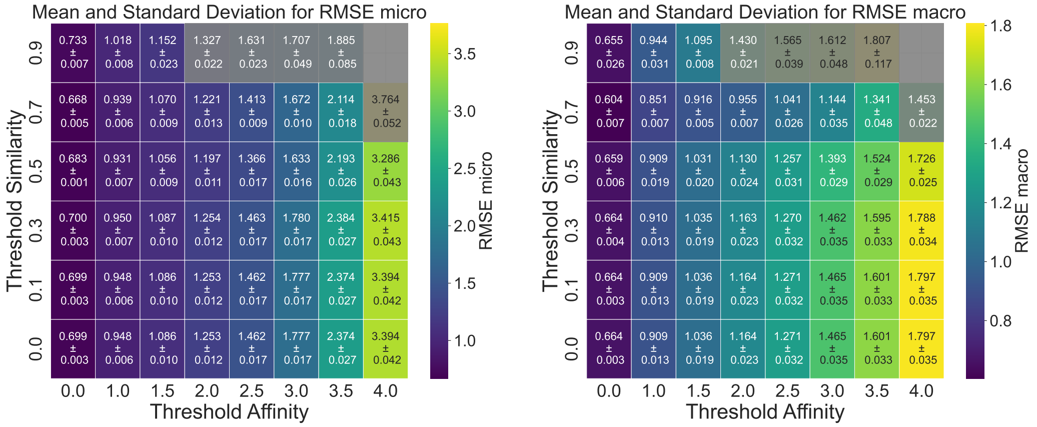

Figure S18: The heatmap of the $RMSE_{micro}$ (left) and $RMSE_{macro}$ (right) of the best DTI model (transfer learning involving both drug and target encoders, **warm start**) for groups of compounds split by similarity and affinity thresholds for KIBA (compound-based split).

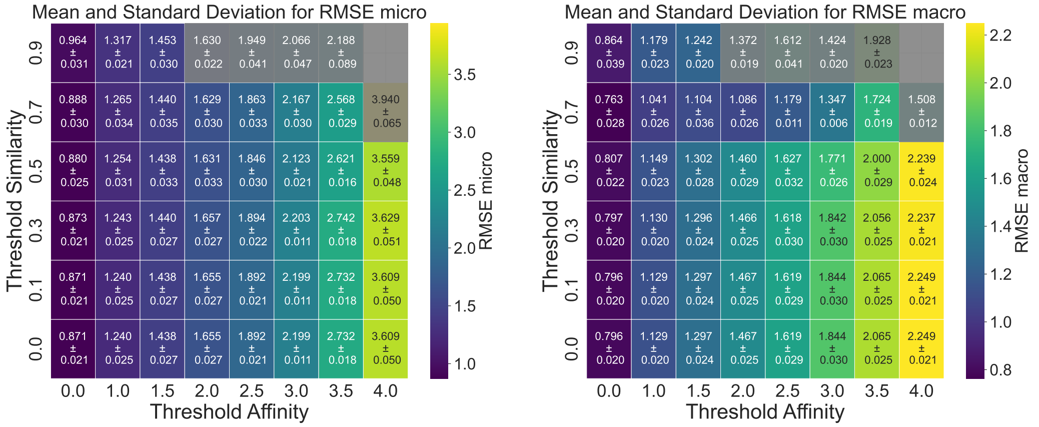

Figure S19: The heatmap of the $RMSE_{micro}$ (left) and $RMSE_{macro}$ (right) of the best DTI model (transfer learning involving both drug and target encoders, **with freezing weights**) for groups of compounds split by similarity and affinity thresholds for KIBA (compound-based split).

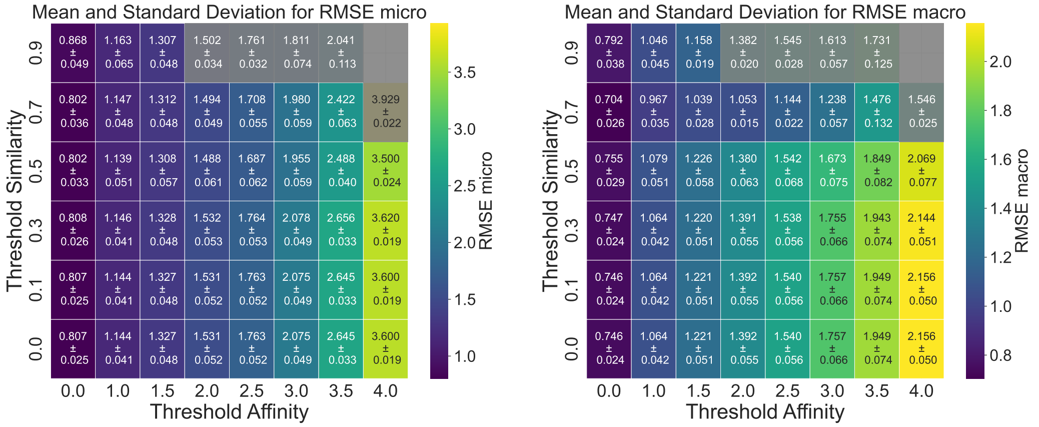

Figure S20: The heatmap of the $RMSE_{micro}$ (left) and $RMSE_{macro}$ (right) of the best DTI model (transfer learning involving both drug and target encoders, **with freezing weights and adding an extra layer**) for groups of compounds split by similarity and affinity thresholds for KIBA (compound-based split).

10.2 BindingDB dataset

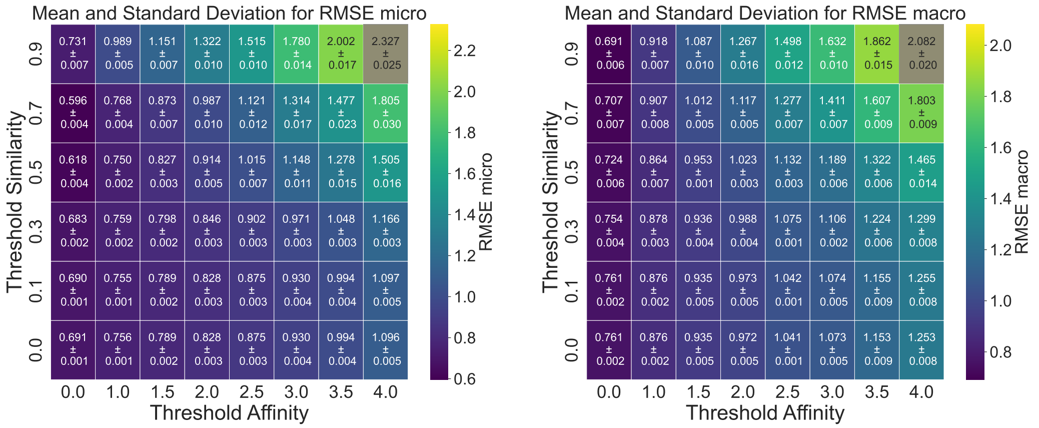

Figure S21: The heatmap of the $RMSE_{micro}$ (left) and $RMSE_{macro}$ (right) of the best DTI model (transfer learning involving both drug and target encoders, **warm start**) for groups of compounds split by similarity and affinity thresholds for BindingDB (random split).

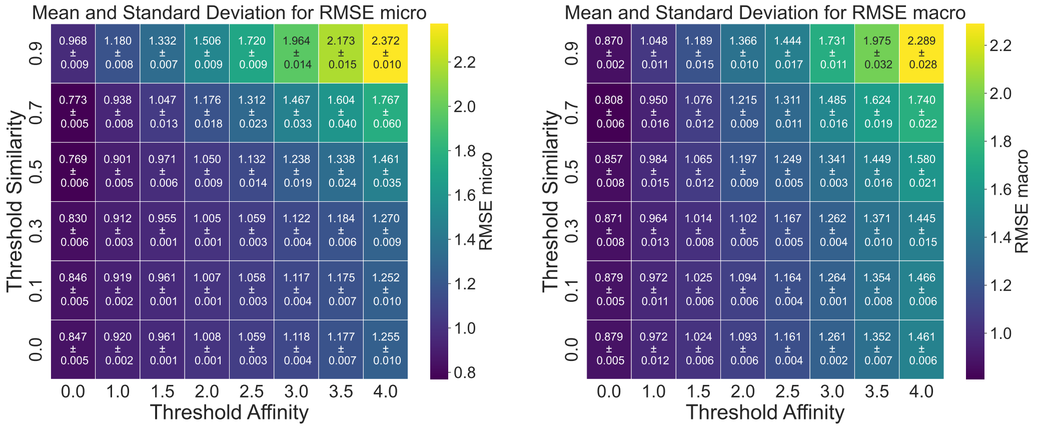

Figure S22: The heatmap of the $RMSE_{micro}$ (left) and $RMSE_{macro}$ (right) of the best DTI model (transfer learning involving both drug and target encoders, **warm start**) for groups of compounds split by similarity and affinity thresholds for BindingDB (compound-based split).

11 Differential heatmaps when transferring only the drug encoder

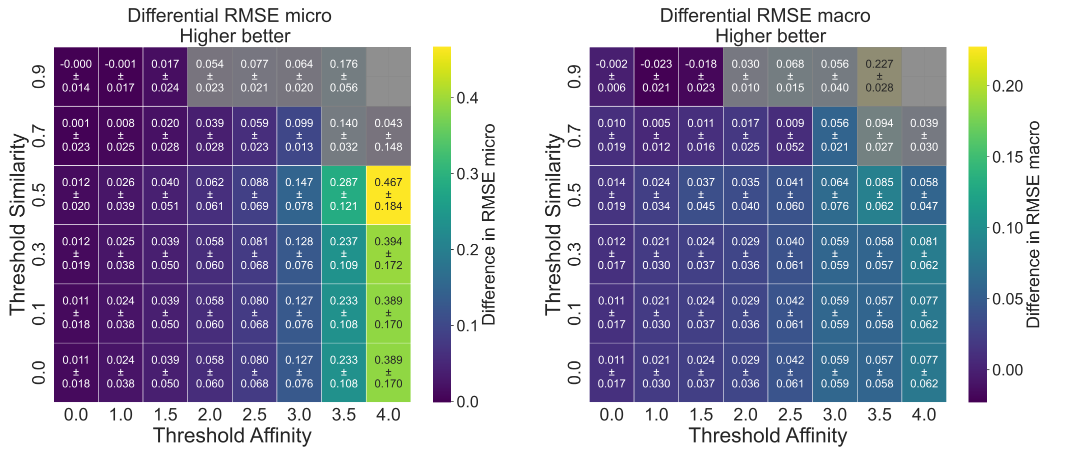

Figure S23: The differential heatmap of the $RMSE_{micro}$ (left) and $RMSE_{macro}$ (right) of the best DTI model (transfer learning involving only the drug encoder, **warm start**) for groups of compounds split by similarity and affinity thresholds for KIBA (random split).

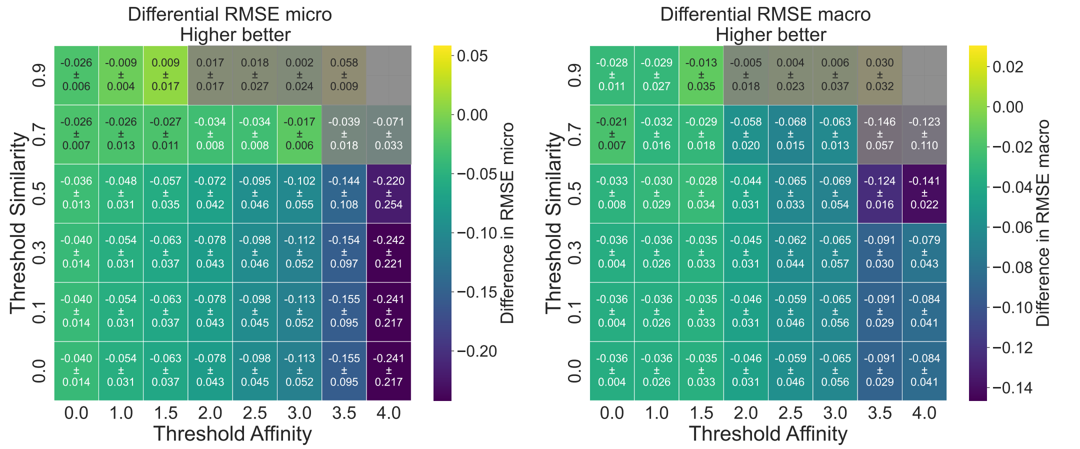

Figure S24: The differential heatmap of the $RMSE_{micro}$ (left) and $RMSE_{macro}$ (right) of the best DTI model (transfer learning involving only the drug encoder, **with freezing weights**) for groups of compounds split by similarity and affinity thresholds for KIBA (random split).

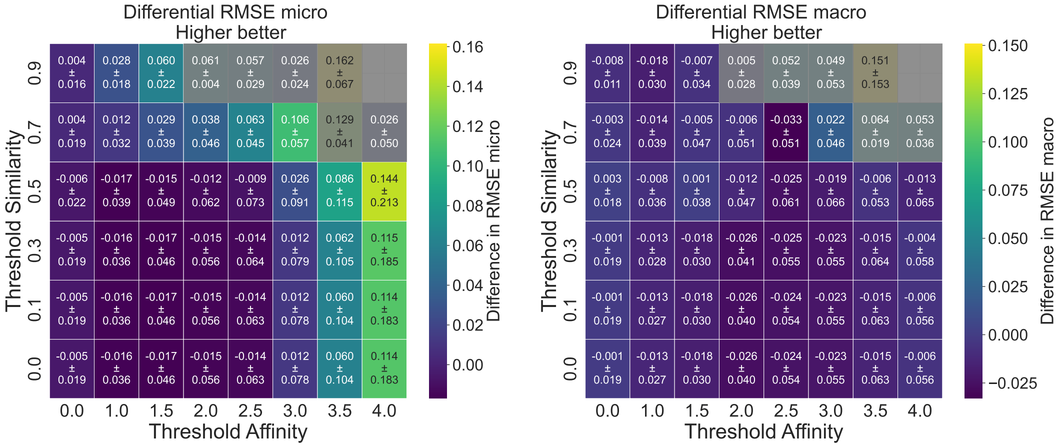

Figure S25: The differential heatmap of the $RMSE_{micro}$ (left) and $RMSE_{macro}$ (right) of the best DTI model (transfer learning involving only the drug encoder, **with freezing weights and adding an extra layer**) for groups of compounds split by similarity and affinity thresholds for KIBA (random split).

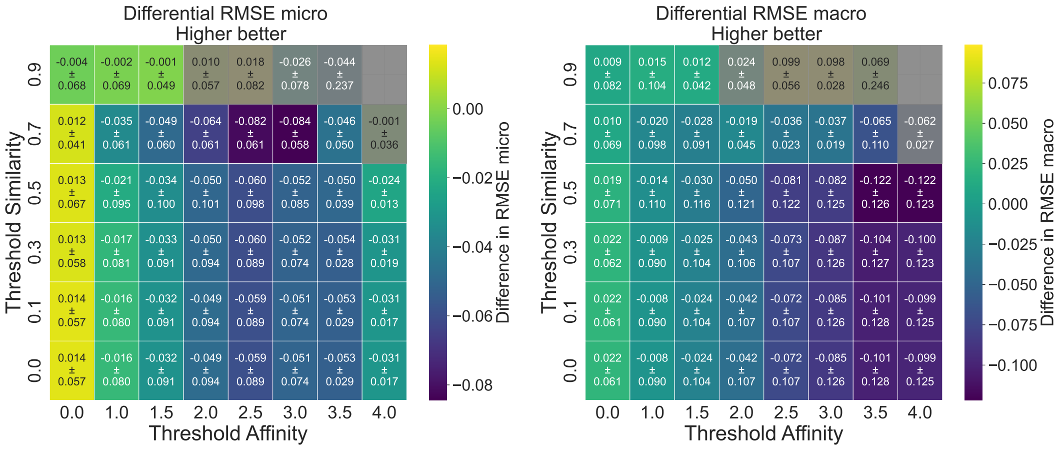

Figure S26: The differential heatmap of the $RMSE_{micro}$ (left) and $RMSE_{macro}$ (right) of the best DTI model (transfer learning involving only the drug encoder, **warm start**) for groups of compounds split by similarity and affinity thresholds for KIBA (compound-based split).

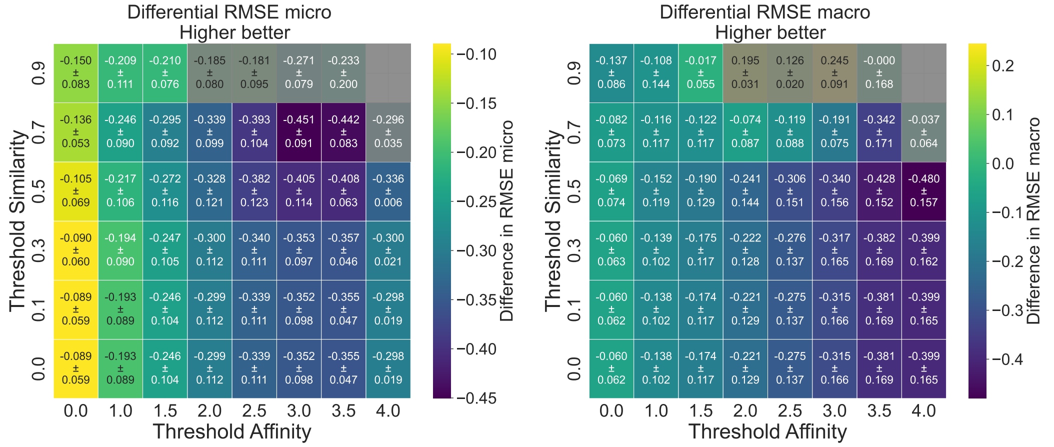

Figure S27: The differential heatmap of the $RMSE_{micro}$ (left) and $RMSE_{macro}$ (right) of the best DTI model (transfer learning involving only the drug encoder, **with freezing weights**) for groups of compounds split by similarity and affinity thresholds for KIBA (compound-based split).

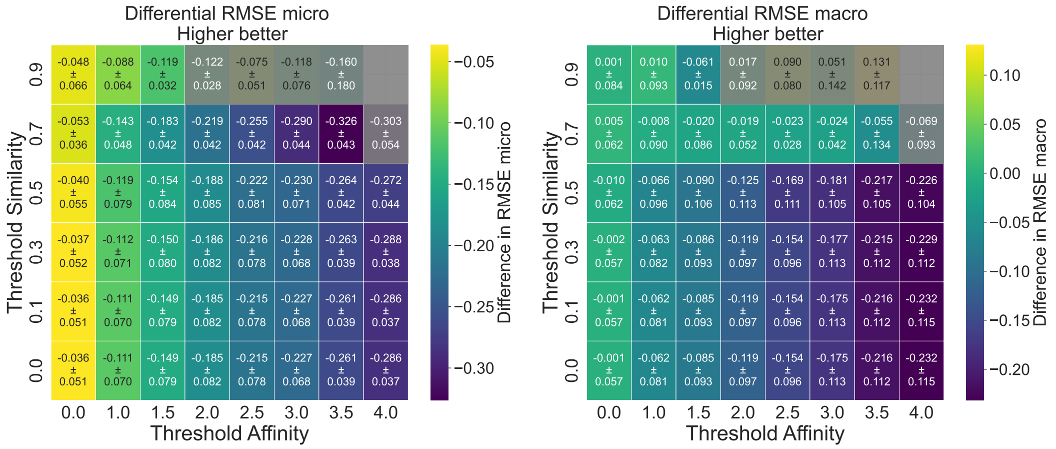

Figure S28: The differential heatmap of the $RMSE_{micro}$ (left) and $RMSE_{macro}$ (right) of the best DTI model (transfer learning involving only the drug encoder, **with freezing weights and adding an extra layer**) for groups of compounds split by similarity and affinity thresholds for KIBA (compound-based split).

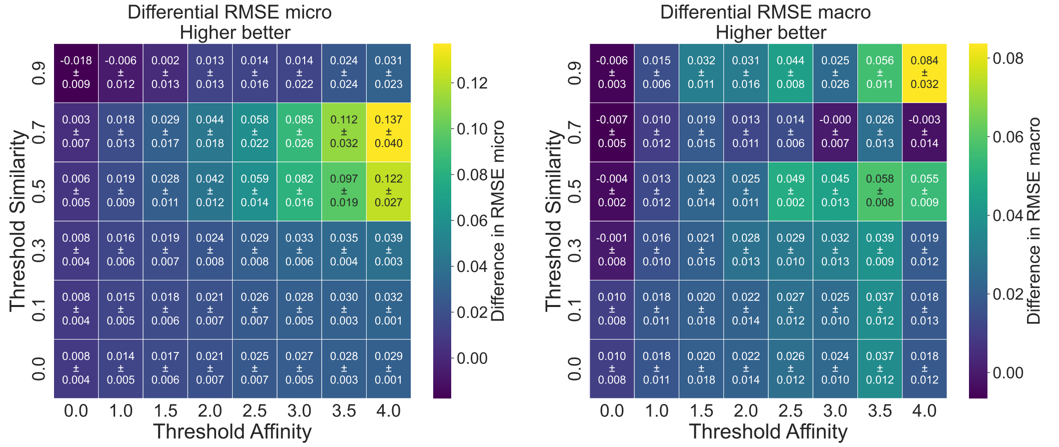

Figure S29: The differential heatmap of the $RMSE_{micro}$ (left) and $RMSE_{macro}$ (right) of the best DTI model (transfer learning involving only the drug encoder, **warm start**) for groups of compounds split by similarity and affinity thresholds for BindingDB (compound-based split).

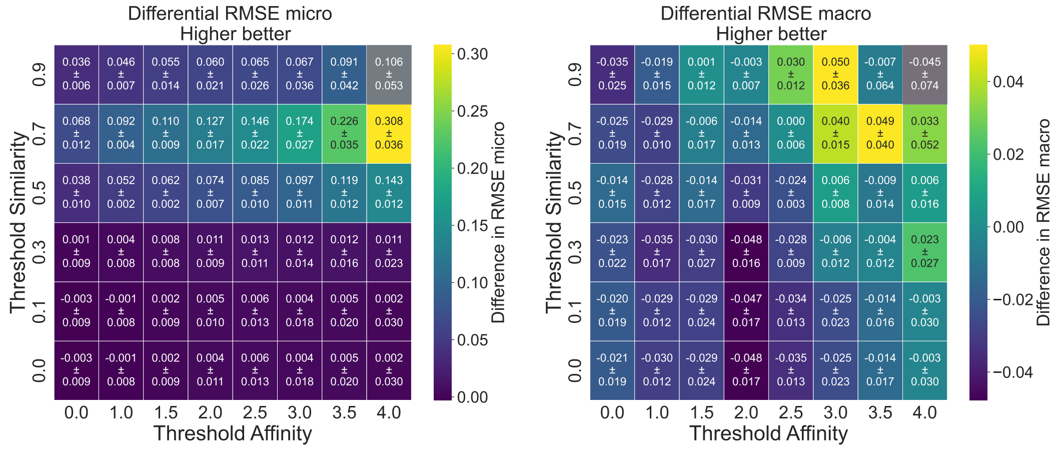

Figure S30: The differential heatmap of the $RMSE_{micro}$ (left) and $RMSE_{macro}$ (right) of the best DTI model (transfer learning involving only the drug encoder, **warm start**) for groups of compounds split by similarity and affinity thresholds for BindingDB (random split).

12 Differential heatmaps when transferring both drug and target encoders

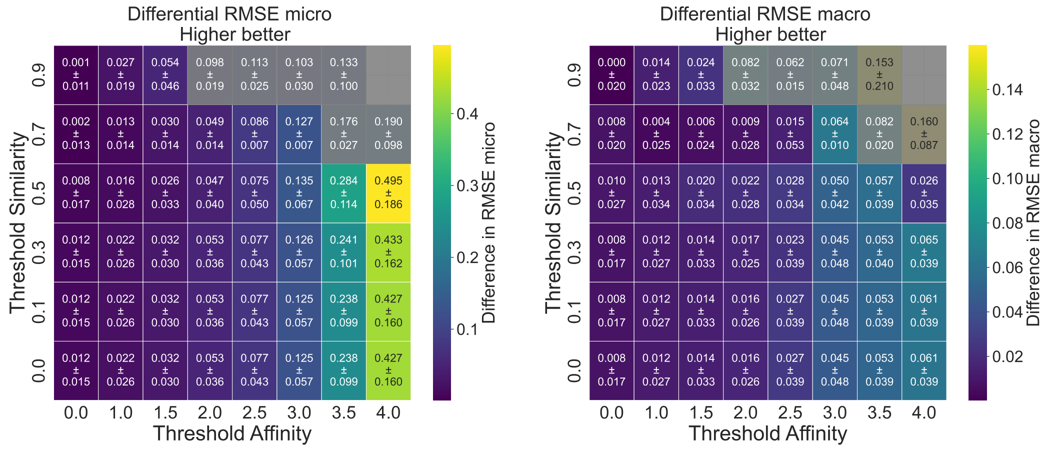

Figure S31: The differential heatmap of the $RMSE_{micro}$ (left) and $RMSE_{macro}$ (right) of the best DTI model (transfer learning involving both drug and target encoders, **warm start**) for groups of compounds split by similarity and affinity thresholds for KIBA (random split).

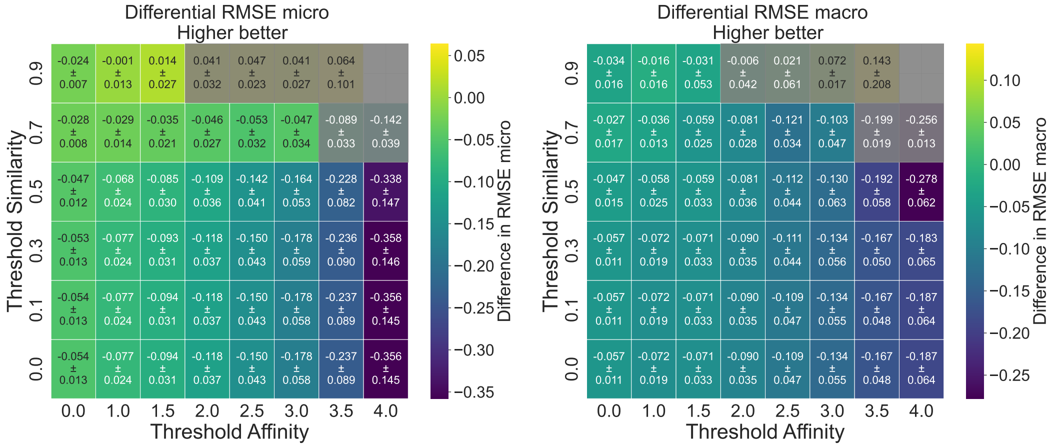

Figure S32: The differential heatmap of the $RMSE_{micro}$ (left) and $RMSE_{macro}$ (right) of the best DTI model (transfer learning involving both drug and target encoders, **with freezing weights**) for groups of compounds split by similarity and affinity thresholds for KIBA (random split).

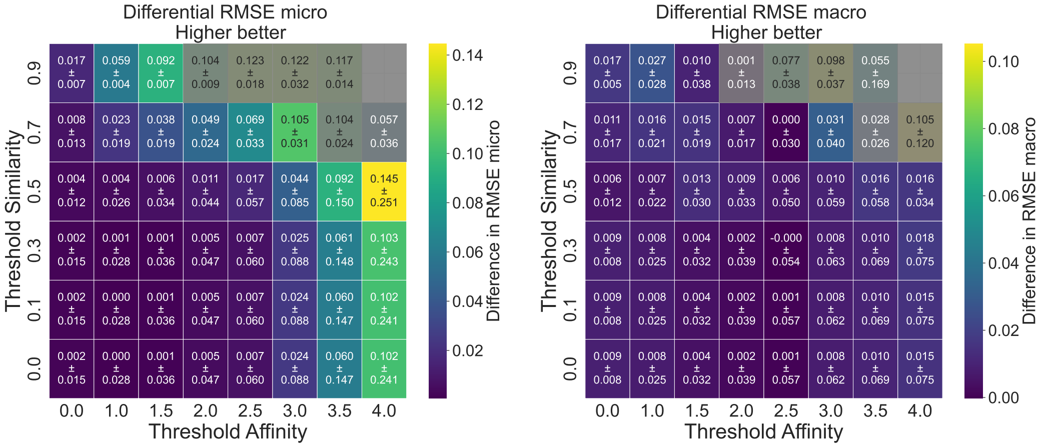

Figure S33: The differential heatmap of the $RMSE_{micro}$ (left) and $RMSE_{macro}$ (right) of the best DTI model (transfer learning involving both drug and target encoders, **with freezing weights and adding an extra layer**) for groups of compounds split by similarity and affinity thresholds for KIBA (random split).

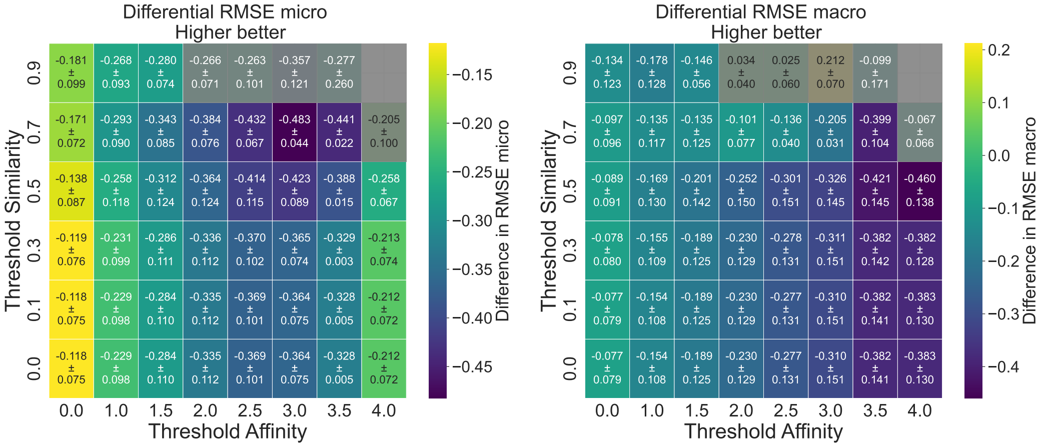

Figure S34: The differential heatmap of the $RMSE_{micro}$ (left) and $RMSE_{macro}$ (right) of the best DTI model (transfer learning involving both drug and target encoders, **with freezing weights**) for groups of compounds split by similarity and affinity thresholds for KIBA (compound-based split).

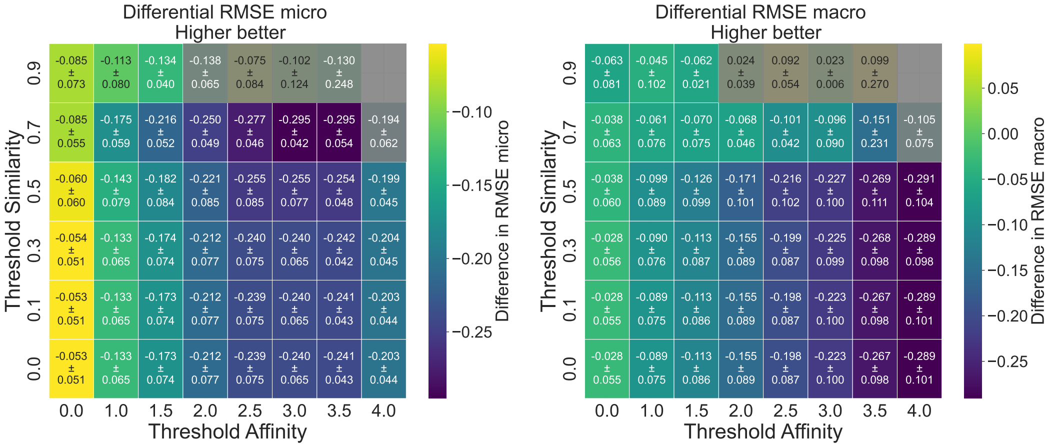

Figure S35: The differential heatmap of the $RMSE_{micro}$ (left) and $RMSE_{macro}$ (right) of the best DTI model (transfer learning involving both drug and target encoders, **with freezing weights and adding an extra layer**) for groups of compounds split by similarity and affinity thresholds for KIBA (compound-based split).

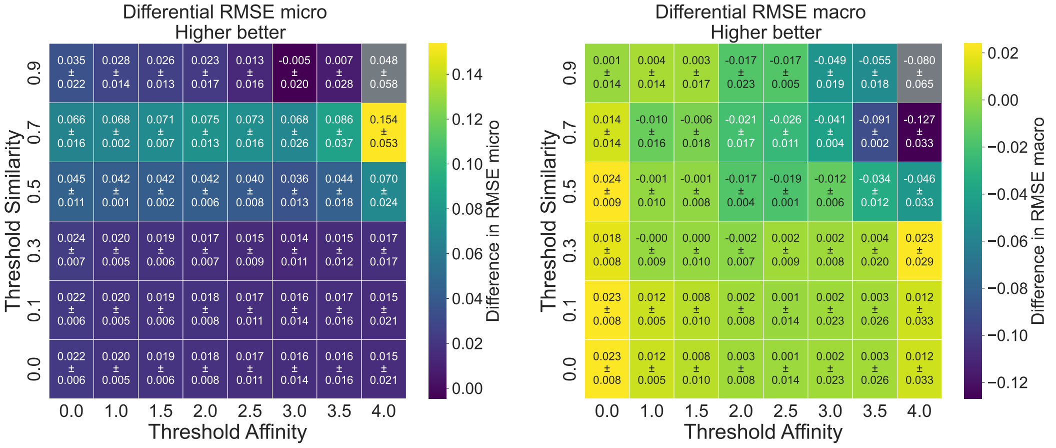

Figure S36: The differential heatmap of the $RMSE_{micro}$ (left) and $RMSE_{macro}$ (right) of the best DTI model (transfer learning involving both drug and target encoders, **warm start**) for groups of compounds split by similarity and affinity thresholds for BindingDB (random split).

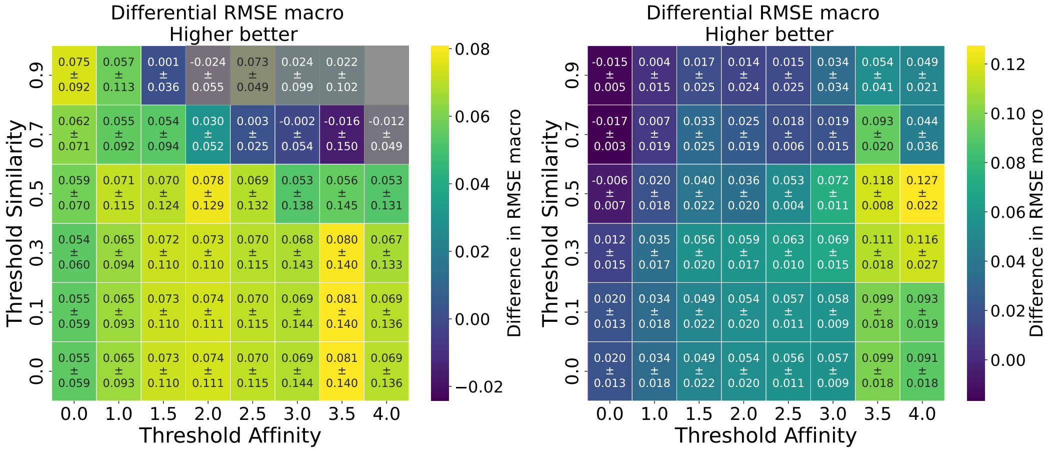

Figure S37: The differential heatmap of the $RMSE_{micro}$ for the best DTI model(transfer learning involving both drug and target encoders, **warm start**) for the KIBA (left) and BindingDB (right) datasets in the case of a compound-based splits, showing groups of compounds split by similarity and affinity thresholds.
